# *Rice stripe virus*-derived siRNAs play different regulatory roles in rice and in the insect vector *Laodelphax striatellus*

**DOI:** 10.1186/s12870-018-1438-7

**Published:** 2018-10-04

**Authors:** Meiling Yang, Zhongtian Xu, Wan Zhao, Qing Liu, Qiong Li, Lu Lu, Renyi Liu, Xiaoming Zhang, Feng Cui

**Affiliations:** 1 State Key Laboratory of Integrated Management of Pest Insects and Rodents, Institute of Zoology, Chinese Academy of Sciences, Bei Chen Xi Lu 1-5, Beijing, 100101 China; 2 Shanghai Center for Plant Stress Biology, Chinese Academy of Sciences, Shanghai, 201602 China; 3 University of Chinese Academy of Sciences, Beijing, 100049 China; 4 Center for Agroforestry Mega Data Science and FAFU-UCR Joint Center for Horticultural Biology and Metabolomics, Haixia Institute of Science and Technology, Fujian Agriculture and Forestry University, Fuzhou, 350002 China

**Keywords:** Rice, *Rice stripe virus*, Small brown planthopper, Virus-derived small interfering RNAs, Small RNA sequencing, Transcriptome

## Abstract

**Background:**

Most plant viruses depend on vector insects for transmission. Upon viral infection, virus-derived small interfering RNAs (vsiRNAs) can target both viral and host transcripts. *Rice stripe virus* (RSV) is a persistent-propagative virus transmitted by the small brown planthopper (*Laodelphax striatellus*, Fallen) and can cause a severe disease on rice.

**Results:**

To investigate how vsiRNAs regulate gene expressions in the host plant and the insect vector, we analyzed the expression profiles of small RNAs (sRNAs) and mRNAs in RSV-infected rice and RSV-infected planthopper. We obtained 88,247 vsiRNAs in rice that were predominantly derived from the terminal regions of the RSV RNA segments, and 351,655 vsiRNAs in planthopper that displayed relatively even distributions on RSV RNA segments. 38,112 and 80,698 unique vsiRNAs were found only in rice and planthopper, respectively, while 14,006 unique vsiRNAs were found in both of them. Compared to mock-inoculated rice, 273 genes were significantly down-regulated genes (DRGs) in RSV-infected rice, among which 192 (70.3%) were potential targets of vsiRNAs based on sequence complementarity. Gene ontology (GO) analysis revealed that these 192 DRGs were enriched in genes involved in kinase activity, carbohydrate binding and protein binding. Similarly, 265 DRGs were identified in RSV-infected planthoppers, among which 126 (47.5%) were potential targets of vsiRNAs. These planthopper target genes were enriched in genes that are involved in structural constituent of cuticle, serine-type endopeptidase activity, and oxidoreductase activity.

**Conclusions:**

Taken together, our results reveal that infection by the same virus can generate distinct vsiRNAs in different hosts to potentially regulate different biological processes, thus reflecting distinct virus-host interactions.

**Electronic supplementary material:**

The online version of this article (10.1186/s12870-018-1438-7) contains supplementary material, which is available to authorized users.

## Background

Many plant viruses, especially persistent-propagative plant viruses, infect a wide range of economical crops, including fruits, vegetables and food crops, representing a serious threat to agriculture production [1]. Approximately 80% of plant viruses depend on insect vectors for transmission [2–4]. This insect dependent transmission can be divided into non-persistent, semi-persistent and persistent types, based primarily on the length of successful transmission of virus to the host plant [5]. Persistent-propagative plant viruses can induce serious disease symptoms in agricultural crops [6], while they generally cause asymptomatic phenotypes in vector insects [7]. Persistent-propagative plant viruses encounter different defense strategies when they replicate in vector insects and host plants [8]. One of the antiviral immunity strategies is the RNA silencing machinery triggered by virus infection. This machinery targets both viral transcripts and host transcripts [9–12]. Virus-derived small interfering RNAs (vsiRNAs) that are involved in the RNAi-based antiviral response can also potentially alter the host transcriptome [13–17]. However, different defense mechanisms mediated by vsiRNAs in host plants and vector insects are poorly documented.

RNA silencing is a widespread antiviral mechanism [5, 18, 19]. vsiRNAs are processed by Dicer from double-stranded RNAs (dsRNAs) or structured single-stranded RNAs (ssRNAs) [20, 21]. In plants, the Dicer like (DCL) protein 4 (DCL4) primarily processes viral RNAs into 21-nt vsiRNAs. DCL2 is responsible for creation of 22-nt vsiRNAs when DCL4 is inactivated or suppressed [22–24]. In contrast, in insects, Dicer-2 and Argonaute 2 (AGO2) have substantial effects on viral replication. Viral dsRNAs accumulate during virus infection and are processed into vsiRNA duplexes by Dicer-2. These vsiRNAs are loaded onto insect AGO2 to target viral transcripts [25–27].

*Rice stripe virus* (RSV) causes rice stripe disease, one of the most destructive rice diseases in Eastern Asia [28, 29]. Since first discovered in 1963 in China, this disease has circulated widely and outbroken many times, causing up to 50% grain loss [30]. In 2004, the infected area in Jiangsu province was 1,571,000 ha, accounting for 80% of the rice field [31]. RSV belongs to the genus *Tenuivirus* and consists of four single-stranded RNA segments that encode seven proteins [20, 32, 33]. RSV is a typical persistent-propagative plant virus and is efficiently transmitted via one of the most economically important insects, the small brown planthopper (*Laodelphax striatellus* Fallen). vsiRNAs were previously identified in RSV-infected *O. sativa, N. benthamiana* and *L. striatellus* [21, 34]. There is large number of RSV-derived sRNAs (vsiRNAs) in these libraries, and of these vsiRNAs, *O. sativa* is the main source followed by *N. benthamiana* and then *L. striatellus.*

However, a comparative functional analysis of RSV vsiRNAs in RSV-infected host plants and vector insects was not reported. To gain further insights into the regulatory functions of vsiRNAs in host plants and an efficient insect vector, we studied the profiles of small RNAs (sRNAs) and mRNAs in RSV-infected rice and small brown planthopper. We show that RSV not only generates vsiRNAs that accumulate in both hosts, but also generates host-specific vsiRNAs that potentially control different defensive responses in the two hosts.

## Results

### sRNA profiles in RSV-infected rice and small brown planthopper

sRNA libraries were constructed and sequenced using sRNAs isolated from rice plants and small brown planthoppers after being infected with RSV for 20 d and 5 d, respectively, with three biological replicates for each group. As controls, mock-inoculated rice and insect samples were prepared and sequenced in parallel. One RSV-infected rice sample and one mock-inoculated rice sample were discarded due to poor quality and only two replicates for each group were retained for analyses. After removal of adapter sequences, and low quality reads, we obtained, on average, 15,400,564, 13,876,277, 15,309,080, and 14,117,003 clean sRNA reads from RSV-infected rice, mock-inoculated rice, RSV-infected planthopper, and mock-inoculated planthopper samples, respectively (Additional file 1 and Additional file 2). After discarding reads mapped to structural RNAs (rRNAs, tRNAs, snoRNAs, snRNAs), the remaining reads were mapped perfectly to the host genome, and 9,208,240, 9,052,920, 6,727,312, and 5,842,694 host-derived sRNAs were obtained on average. These sRNAs were clustered into, on average, 2,468,202, 2,235,158, 1,330,659 and 1,141,043 unique sRNA reads.

**Fig. 1:**
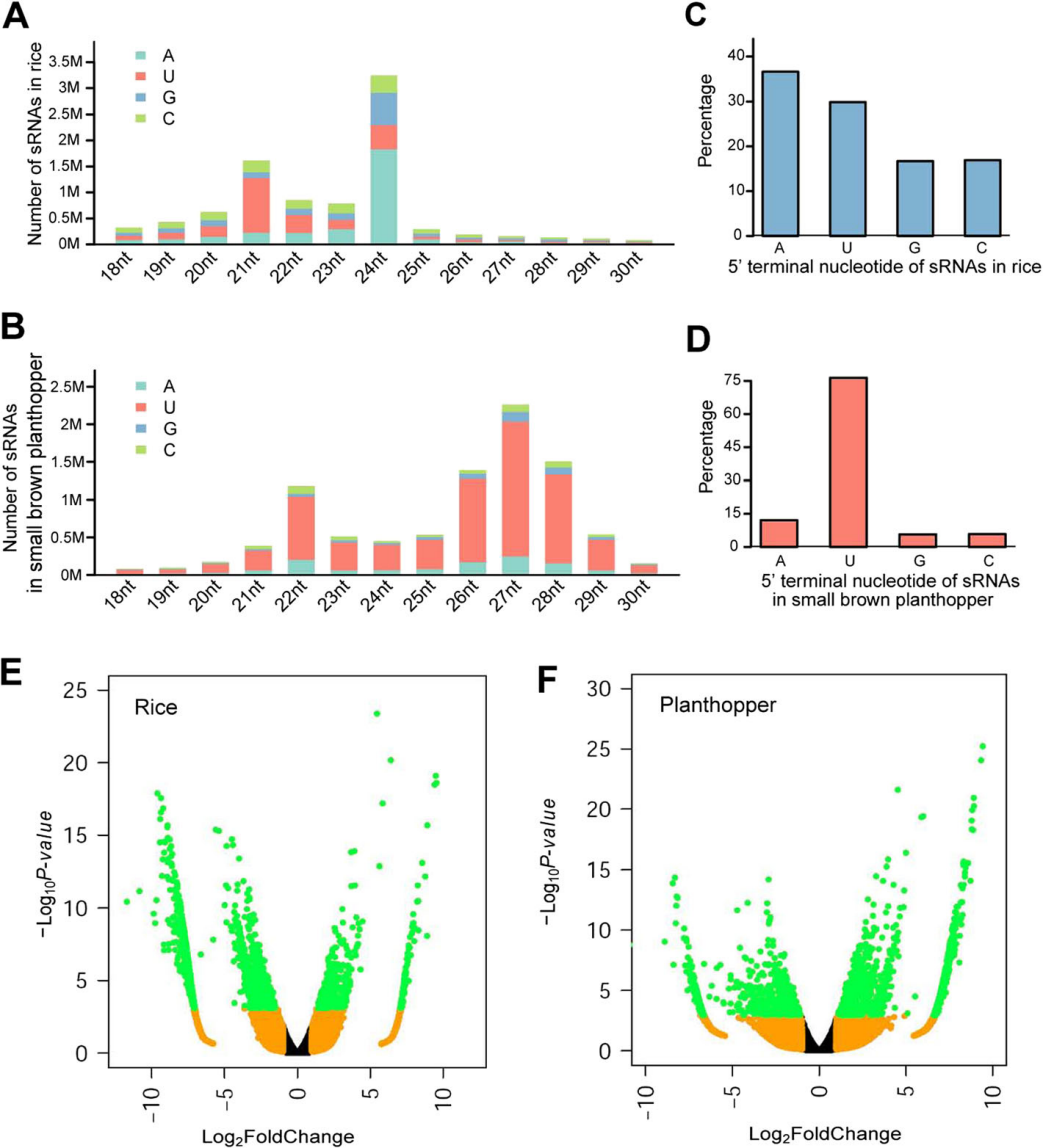
Host-derived sRNAs in rice and small brown planthopper. (**a**, **b**) Size distribution of host-derived sRNAs in rice (**a**) and small brown planthopper (**b**). Host-derived sRNAs are 18–30-nt sRNAs with perfect match to the host genome minus those derived from structural RNAs. Numbers of sRNAs of the same size but with different 5′ terminal nucleotides were drawn in different colors. **c**, **d** 5′ terminal nucleotide frequency of host-derived sRNAs in rice (**c**) and small brown planthopper (**d**). **e**, **f** Volcano plot of differentially expressed sRNAs between mock-inoculated and RSV infected samples in rice (**e**) and small brown planthopper (**f**). Green dots represent differentially expressed unique sRNAs with significance cutoffs being fold change > 2 and adjusted *p*-value < 0.05

The size distributions were quite different for rice- and insect-derived sRNAs. For rice sRNAs, the dominant sizes were 21-nt and 24-nt (Fig. 1a and Additional file 3), whereas the dominant sizes of sRNAs in planthopper were 22-nt and 26-nt to 28-nt (Fig. 1b and Additional file 3), reflecting different sRNA processing properties in these two hosts. The GC contents of sRNAs of different sizes in rice and planthopper samples were calculated. Rice-derived sRNAs had a higher average GC content than did insect-derived sRNAs (Additional file 4).

Because the 5′ terminal nucleotides of sRNAs are important for AGO sorting and function, we analyzed the 5′ nucleotide composition of sRNAs in each library. In rice, uridine (U) or adenosine (A) was the dominant 5′ terminal nucleotide for the 21-nt and 24-nt sRNAs, respectively (Fig. 1a and Additional file 3). In planthopper, U was the dominant nucleotide at the 5′ terminus for the 22-nt and 26-nt to 28-nt sRNAs (Fig. 1b and Additional file 5). In contrast, U and A were the most abundant 5′ terminal nucleotides for rice total sRNAs, while U was still the dominant 5′ terminal nucleotide for planthopper total sRNAs (Fig. 1c, d and Additional file 6).

We compared the abundance of host sRNAs in RSV-infected samples and mock-inoculated samples to identify host sRNAs that were regulated upon RSV infection. Compared to the mock-inoculated samples, 450 and 1,558 sRNAs were up- and down-regulated in RSV-infected rice, and 1,776 and 783 sRNAs were up- and down-regulated in RSV-infected planthopper (Fig. 1e and f).

### RSV-derived siRNAs in RSV-infected rice and small brown planthopper

**Fig. 2:**
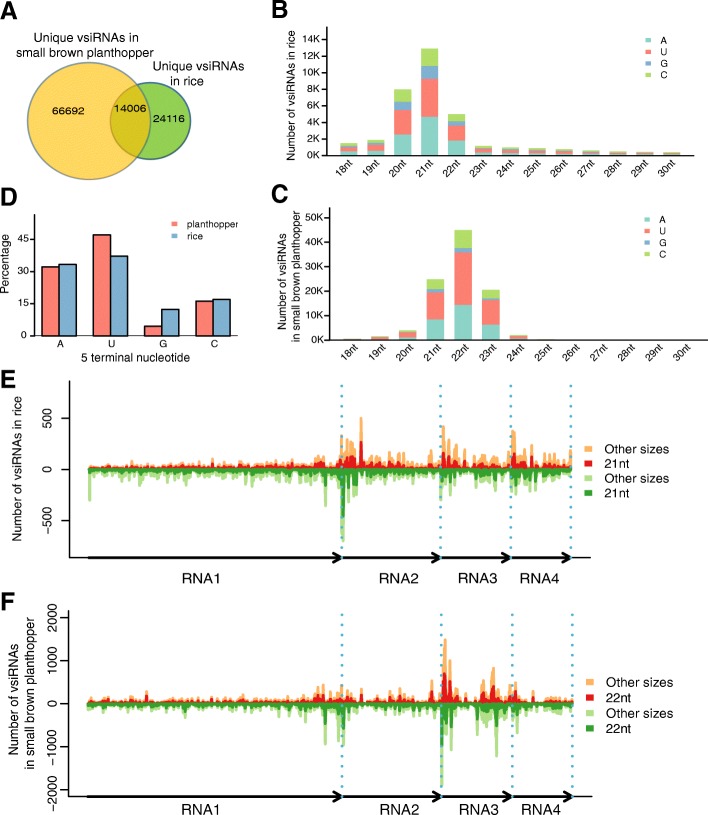
vsiRNAs in rice and small brown planthopper. **a** Venn diagram showing number of unique vsiRNAs that were specific to rice or planthopper and those found in both hosts. **b**, **c** Size distribution of vsiRNAs in rice (**b**) and small brown planthopper (**c**). vsiRNAs are 18–30-nt sRNAs in RSV-infected rice and planthopper with up to one mismatch to the RSV genome. Numbers of vsiRNAs of the same size but with different 5′ terminal nucleotides are drawn in different colors. **d** 5′ terminal nucleotide frequency of vsiRNAs in RSV-infected rice and planthopper. **e**, **f** Distribution of vsiRNAs along the RSV genome in RSV-infected rice (**e**) and small brown planthopper (**f**). Four segments of the RSV genome were arranged along the X-axis with length drawn to scale. vsiRNAs that were derived from the sense and antisense strand were shown above and below the horizontal line, respectively. Numbers of vsiRNAs with the most abundant size class (21-nt in rice and 22-nt in planthopper) were separated from those of other size classes

We mapped clean sRNA reads to the RSV genome and obtained RSV-derived siRNAs. We identified 38,122 and 80,698 unique vsiRNAs in RSV-infected rice and viruliferous planthopper, respectively, and 14,006 unique vsiRNAs were found in both hosts (Fig. 2a). In RSV-infected rice, the size of vsiRNAs ranged from 18-nt to 30-nt, with a peak at 21-nt (Fig. 2b, Additional file 7 and Additional file 8). In RSV-infected planthoppers, the size of vsiRNAs ranged predominantly from 18-nt to 24-nt, with a peak at 22-nt (Fig. 2c, Additional file 7 and Additional file 8). In addition, the preferential occurrence of A/U at the 5′ terminus was identified for the vsiRNAs in rice and planthoppers (Fig. 2d and Additional file 9).

We also looked in detail at the strand specificity and locations of origin of vsiRNAs. As shown in Fig. 2e, f and Additional file 10, the proportions of vsiRNAs that were generated from sense and antisense genome sequences were comparable in both hosts. Regarding the genomic regions from which vsiRNAs were generated, far fewer vsiRNAs were produced from RNA1 than from the other three RNA segments in rice (Fig. 2e). In contrast, RNA3 generated more vsiRNAs than the other three segments in planthopper (Fig. 2f). Moreover, accumulation peaks in the termini of viral RNA segments were observed in rice, but the vsiRNAs were more evenly distributed in planthopper (Fig. 2e, f).

**Fig. 3:**
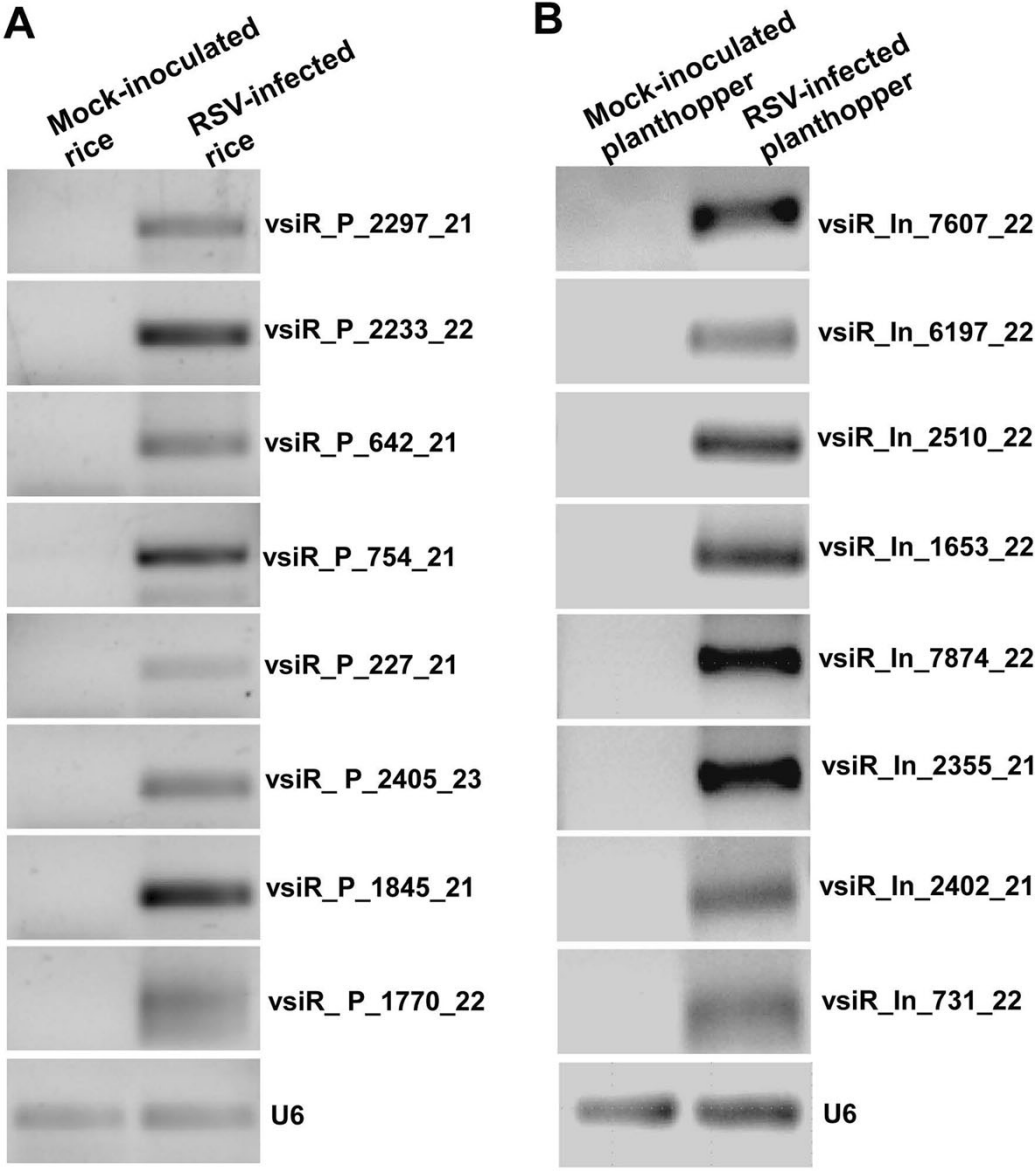
RT-PCR validation of the expressions of vsiRNAs in RSV-infected rice (**a**) and small brown planthopper (**b**). RT-PCR assays were conducted with three independent biological replicates. U6 was used as internal control

The 8 most abundant vsiRNAs in rice and planthopper were selected for RT-PCR validation, respectively. The results showed that these vsiRNAs accumulated highly in RSV-infected rice/planthopper, demonstrating that RSV infection indeed generated highly abundant vsiRNAs in both host plants and vector insects (Fig. 3).

### Transcriptome alterations of rice and planthopper upon RSV infection

To explore possible regulation mechanisms mediated by vsiRNAs in rice and small brown planthopper, we analyzed their transcriptomic responses to RSV infection. The same samples used for sRNA sequencing were subjected to mRNA sequencing and differentially expressed genes (DEGs) between RSV-infected and mock-inoculated samples were identified.

**Fig. 4:**
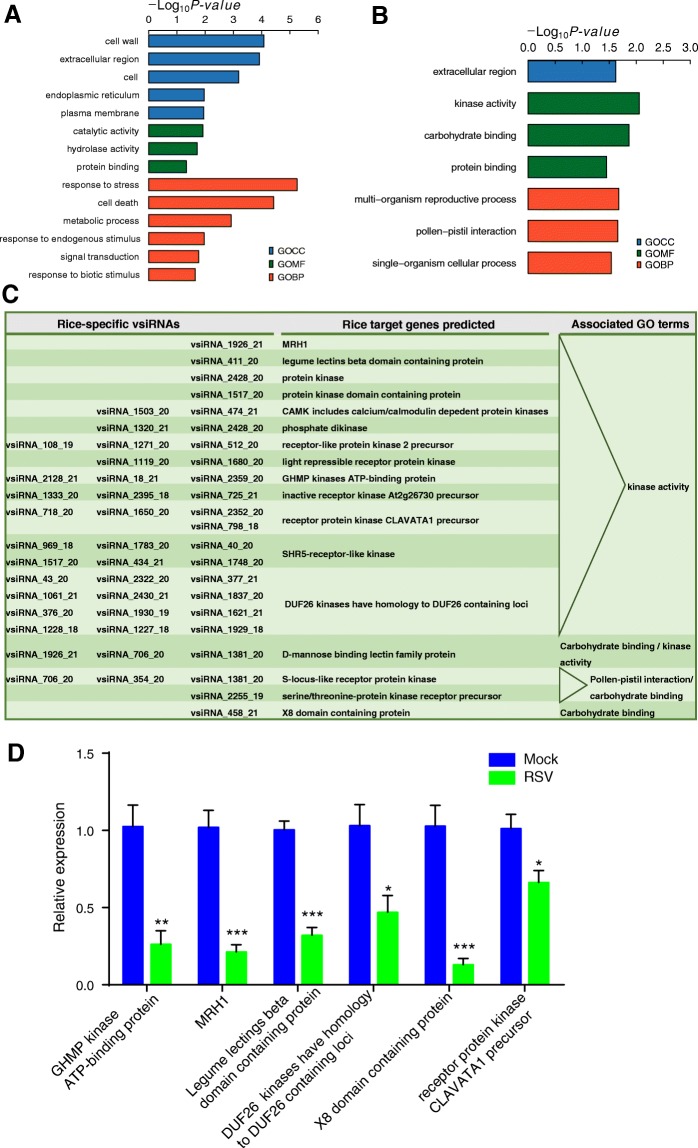
Rice genes that are potentially targeted by rice-specific vsiRNAs. **a** GO enrichment analysis of the down-regulated genes in rice after RSV infection. **b** GO enrichment analysis of the down-regulated genes that are potentially targeted by vsiRNAs. **c** Selected rice-specific vsiRNAs that potentially interact with rice genes involved in kinase activity, carbohydrate binding and pollen-pistil interaction. **d** Six predicted target genes of vsiRNA were selected to verify downregulation using qRT–PCR. qPCR data are shown as the means ± SEM (*n* = 6). *p < 0.05; **p < 0.01; ***p < 0.001

After infection with RSV, a total of 989 DEGs were identified in rice, among which 273 genes were down-regulated (Additional file 11 and Additional file 12). GO term enrichment analysis showed that 14 GO terms were enriched for the down-regulated genes in rice, including: response to stress, cell death, metabolic process, cell wall, extracellular region and cell as the most significantly enriched (*p*-value < 0.01) (Fig. 4a). Furthermore, 48 down-regulated genes were potential targets of vsiRNAs according to sequence complementarity. These target genes were enriched with genes involved in: kinase activity (*p*-value < 0.01), carbohydrate binding, protein binding, extracellular region, multi-organism reproductive process, pollen-pistil interaction, and single-organism cellular process (*p*-value < 0.05) (Fig. 4b). In the 716 up-regulated genes of rice, the most significantly enriched GO terms were response to biotic stimulus or stress, metabolic process, oxygen binding, catalytic activity and carbohydrate binding (*p*-value < 0.01) (Additional file 13).

**Fig. 5:**
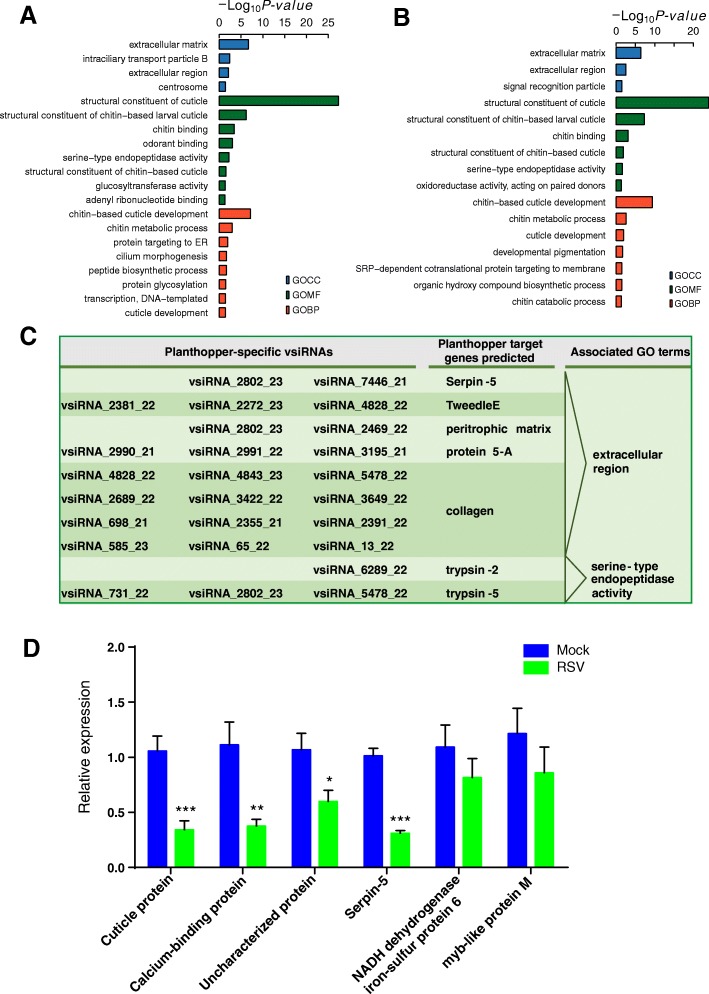
Planthopper genes that are potentially targeted by planthopper-specific vsiRNAs. (A) GO enrichment analysis of the down-regulated genes in small brown planthopper after RSV infection. (B) GO enrichment analysis of the down-regulated genes that are potentially targeted by vsiRNAs. (C) Selected planthopper-specific vsiRNAs that potentially target planthopper genes encoding secretion proteins or involved in serine-type endopeptidase activity. (D) Six predicted target genes of vsiRNA were selected to verify downregulation using qRT–PCR. qPCR data are shown as the means ± SEM (n = 6). *p < 0.05; **p < 0.01; ***p < 0.001

In RSV-infected planthopper, 551 DEGs were identified, among which 265 genes were down-regulated (Additional file 11 and Additional file 14). 20 GO terms were enriched for the down-regulated genes: extracellular matrix and region, structural constituent of chitin-based larval cuticle, chitin binding, odorant binding, chitin-based cuticle development, and chitin metabolic process (Fig. 5a).

Seventy down-regulated genes were predicted to be the targets of vsiRNAs. These target genes were enriched in genes involved in: cuticle-related molecular functions or processes, signal recognition particle, extracellular matrix and region, oxidoreductase activity acting on paired donors, developmental pigmentation, SRP-dependent cotranslational protein targeting to membrane, and organic hydroxy compound biosynthetic process (Fig. 5b). In the 286 up-regulated genes, genes that were significantly enriched were involved in: response to gap junction, iron ion binding, cellular calcium ion homeostasis, and hexose catabolic process (Additional file 15).

### Host-specific vsiRNAs targeted kinase activity genes in rice and pathogen/disease resistance genes in planthoppers

We then investigated the targets of host specific vsiRNAs in rice and planthoppers. Among the 48 potential targets of vsiRNAs in rice, 17 genes were found to be targets of rice specific vsiRNAs (Fig. 4c and Additional file 16). Thirteen out of the 17 genes had a kinase activity, such as protein kinases and receptor protein kinases. The other four potential targets took part in carbohydrate binding and pollen-pistil interaction, such as D-mannose binding lectin family protein and serine/threonine-protein kinase receptor precursor. These 17 genes were predicted to be targeted by from one to three rice-specific vsiRNAs (Fig. 4c). Six of these 17 genes were selected to validate differential expression patterns between RSV-infected and mock-inoculated rice using qRT-PCR. All of the 6 genes showed significantly lower transcript levels in RSV-infected rice than in mock-inoculated rice (Fig. 4d). These results are consistent with the transcriptome data.

In the 70 potential targets of vsiRNAs in planthoppers, 6 genes were found to be targets of planthopper specific vsiRNAs (Fig. 5c and Additional file 17). Some of these encoded pathogen/disease resistance proteins, such as trypsins, serpin-5, and peritrophic matrix protein (Fig. 5c). These 6 genes were regulated by from one to three planthopper specific vsiRNAs. Among the 70 genes, we investigated 6 potential targets, among which 4 had lower transcript levels in RSV-infected planthoppers and 2 had no significant differences between RSV-infected and mock-inoculated groups (Fig. 5d).

### Common vsiRNAs regulated different targets in rice and planthopper

**Fig. 6:**
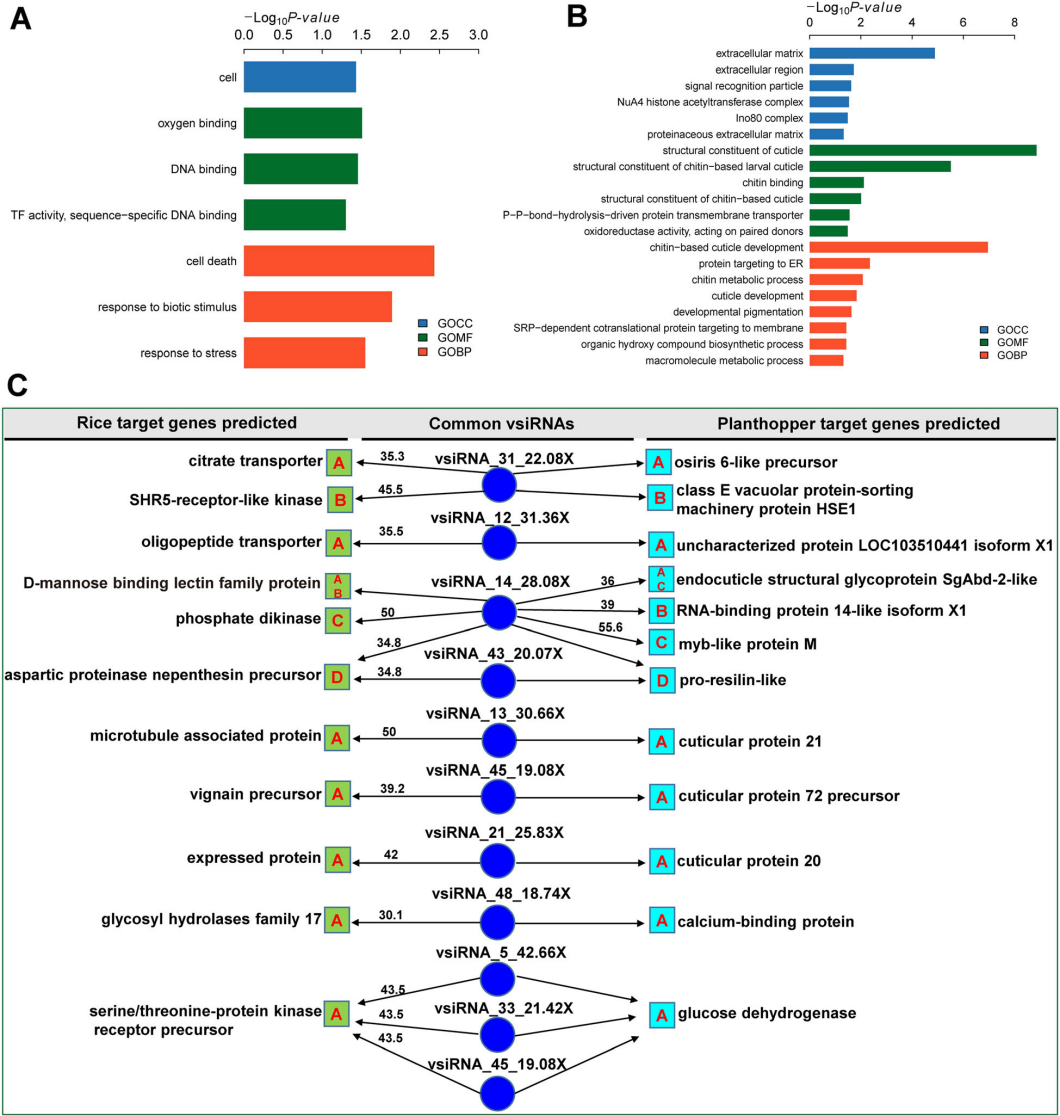
Common vsiRNAs in RSV-infected rice and planthopper potentially target genes with different functionalities. **a**, **b** Gene ontology (GO) enrichment analysis of the down-regulated genes that were potentially targeted by the 50 most highly expressed common vsiRNAs in RSV-infected rice (**a**) and viruliferous planthopper (**b**). **c** Target genes of common vsiRNA in the two hosts that show sequence homology. Blue circles represents common vsiRNAs. Black arrows represent vsiRNA-target relationship. Colored boxes with “A”-“D” labels represent predicted target genes. The genes targeted by the same vsiRNA in the two hosts are labeled by the same letter because they show sequence homology, the level of which is indicated above the black arrow

To determine whether same vsiRNAs can regulate similar pathways in host rice and vector planthopper upon RSV infection, we selected the 50 most abundant vsiRNAs from the 14,006 vsiRNAs that were expressed in rice and planthopper to predict their potential targets. A total of 39 and 58 down-regulated genes were predicted to have potential binding sites for one or more of the 50 vsiRNAs in rice and planthopper, respectively. GO analysis of the 39 predicted rice target genes showed that many of them encoded immune response and defense proteins that are involved in cell death and response to stress and biotic stimulus (Fig. 6a). In contrast, the 58 predicted planthopper target genes were most enriched in GO terms of extracellular matrix, structural constituent of cuticle, structural constituent of chitin−based larval cuticle, and chitin−based cuticle development (Fig. 6b). Therefore, it appears that RSV vsiRNAs may manipulate distinct biological processes in host plant and vector insect. To uncover whether vsiRNAs can target evolutionarily conserved genes in these two hosts, we used the 39 rice and 58 planthopper potential target genes as queries to search for homologous genes in the other host. We found that 11 rice target genes showed > 30% identity at the amino acid level with 12 planthopper target genes. However, these homolog-like target genes did not have similar functional annotations (Fig. 6c), indicating that they may perform different functions.

## Discussion

In this paper, we have examined the expression profiles of vsiRNAs in RSV-infected rice and planthopper, and showed that RSV generated either same or distinct vsiRNAs, potentially regulating different biological processes in the two hosts. A few studies have shown that vsiRNAs, as pathogenicity determinants, negatively regulate host mRNAs and effectively silence host genes [15, 35–40]. For example, chloroplast-related genes can be targeted by vsiRNA to regulate the symptoms of twisted-leaves and stunting [32]. Our study suggests that a given virus can generate distinct vsiRNAs in different hosts to regulate different biological processes, reflecting distinct virus-host interactions.

The mechanisms of vsiRNA production may be different in plant and insect. We found that the 21-nt vsiRNA was the main size-class of vsiRNAs in rice while the 22-nt vsiRNA turned to be the most abundant class of vsiRNAs in planthopper, which was consistent with the findings from another study [21]. Different sizes of vsiRNAs may be processed by different Dicers/DCLs. DCL4 is known to be mainly responsible for production of 21-nt vsiRNAs and DCL2 is responsible for the production of 22-nt vsiRNAs for rescuing silence against RNA viruses when DCL4 was inactivated or suppressed [22–24, 41]. Further investigation is needed to elucidate the role of rice DCL4 and DCL2 in producing RSV vsiRNAs.

Our study revealed a preferential occurrence of uridine or adenosine residues, as compared to cytosine and guanidine, at the 5′ terminus of vsiRNAs in rice and planthopper. Previous studies have indicated that the 5′-terminal nucleotides of sRNAs control the sorting of sRNAs to specific AGO complexes in plant [42, 43]. In *Drosophila*, sRNAs were sorted for AGO1 or AGO2 according to their duplex structures and 5′-terminal nucleotides [44, 45]. The 5′-terminal nucleotides of vsiRNAs from rice and planthopper were similar, suggesting that these nucleotides of 5′-terminal may have role(s) in loading of vsiRNAs into specific AGO complexes.

The hot spots of RSV-derived vsiRNA in rice are predominantly at the terminal regions of the RSV RNA segments. This phenomenon is similar to that of *Cucumber mosaic virus* (CMV) vsiRNAs, which are also enriched in the terminal regions of CMV genomes. Moreover, this enrichment is lost in RNA-dependent RNA polymerases (*rdr*s) mutant plants infected with CMV. Thus RDRs are supposed to be involved in amplification of vsiRNAs from CMV genomic RNAs [46]. The enrichment of RSV-derived vsiRNAs in the terminal regions in rice may also originate from the host RDR-mediated vsiRNAs amplification.

We find that rice-derived sRNAs tend have a higher average GC content than do insect-derived sRNAs. The sRNA structural features of different GC values may affect accessibility, stability, or activity of the components in sRNAs biogenesis pathway. Higher GC content around the sRNA generating site may result in forming a relatively more stable secondary structure, which would be a preferable substrate for DCL processing [47]. The difference of GC content of insect-derived sRNAs and rice-derived sRNAs point to the different sRNAs biogenesis pathway in insect and rice.

vsiRNA acts as an effective cross-talk molecule to defend the stress signaling components of hosts after RSV infection. The potential cross reaction of vsiRNAs with host genes is a systematic counter-offensive strategy deployed by virus to shift the virus/host ‘tug of war’ in the pathogen’s favor. Some pathogen/disease resistance genes such as genes encoding trypsins and peritrophic matrix protein are down-regulated by vsiRNAs in planthopper [48, 49]. The down-regulation of these resistance genes would favor RSV infection and replication in the planthopper. In rice, some crucial genes, such as those encoding protein kinases, mannose binding lectin family protein, act as hubs in the regulatory network in plant response to abiotic and biotic stress. Protein kinases are involved in defense of different plant virus infections in tomato and tobacco [50–53]. Mannose binding lectin genes are critical in developing resistance to various pathogenic organisms and insects [54–57]. Down-regulation of genes encoding protein kinases and mannose binding lectin family proteins by vsiRNAs may facilitate RSV pathogenicity in rice.

Some vsiRNA-derived sRNA common in host rice and vector planthopper were found to potentially target one or several host factors involved in various physiological pathways of different host. Similarly, vsiRNA derived from grapevine fleck virus (GFkV) and Grapevine rupestris stem pitting-associated virus (GRSPaV) were shown to target *Vitis vinifera* derived genes involved in ribosome biogenesis, biotic and abiotic stresses besides their role in plant’s defense mechanism [36]. Accordingly, the targets of the corresponding common vsiRNAs from both host plant and vector insect are diverse not only in sequence complementarity but on the positions of the vsiRNA targets in mRNA, secondary structural features of the target mRNA [10], which could also play a significant role in small RNA-targeted gene silencing.

## Methods

### Virus, vector and host plant preparation

The viruliferous and non-viruliferous small brown planthopper strains used in this work were derived from a rice field population in Hai’an, Jiangsu Province, China, in 2009, and maintained in the laboratory until now. The lengthy period of maintenance and screening make sure that no other pathogens exist in the planthopper strains. The planthoppers were reared on 2 cm to 3 cm seedlings of rice (*Oryza sativa* Huangjinqing) in glass incubators, which were sealed with a nylon mesh, at 25 ± 1 °C, 80% relative humidity, and 16 h of light per day. The planthoppers were transferred to fresh rice seedlings every 8 days to ensure sufficient nutrition. The RSV-carrying rate of the viruliferous planthopper strains were maintained at no less than 90% through a purification selection every three months using a dot-ELISA assay with the monoclonal anti-Cp antibody [58].

### Virus inoculation

Viruliferous fourth-instar planthoppers were transferred to fresh rice seedlings for RSV inoculation. After 24 h feeding, the planthoppers were removed and the inoculated seedlings were cultured for 20 d until disease symptom appeared. Each rice seedling was fed by three viruliferous planthoppers, and three seedlings were used in a replicate experiment. Healthy rice seedlings without inoculation were prepared and maintained at the same condition as the negative control. Leaves with typical disease symptoms from RSV-infected rice plants and leaves from healthy rice plants were collected at the same time and three biological replicates for each group were prepared for sequencing analysis.

Non-viruliferous fourth-instar planthopper nymphs were fed on an artificial diet containing the RSV crude preparations from the infected rice leaves as previously described [59]. After feeding on RSV for 8 h, the nymphs were transferred to healthy rice seedlings and then collected after 5 d. Three biological replicates and 20 insects per replicate were prepared for sequencing analysis.

### RNA isolation and sequencing

Total RNA was isolated from rice plants or planthoppers using the TRIzol Reagent (Invitrogen, Carlsbad, CA, USA) according to manufacturer’s instructions. Approximately 100 mg of rice leaves or 10 small brown planthoppers were ground in liquid nitrogen and mixed with 1 ml of TRIzol Reagent for RNA extraction. The concentration and quality of RNA were measured using NanoDrop spectrophotometer (Thermo Scientific, Waltham, MA, USA) and gel electrophoresis. Total RNA was used for mRNA library and sRNA library constructions. mRNA sequencing was performed on an Illumina Hiseq 2500 sequencer to generate paired-end 125 bp data and the sRNA sequencing was conducted on an Illumina Hiseq 4000 sequencer to generate single-end 50 bp sequences in Beijing Genome Institute (BGI, Shenzhen, China).

### Genome datasets

The rice (*Oryza sativa ssp. japonica* cv. Nipponbare) genome sequence (v. 7.0) and its gene annotations, including Gene Ontology (GO) annotations, were downloaded from the Rice Genome Annotation Project (http://rice.plantbiology.msu.edu). The genome sequence of the small brown planthopper and GO annotations have been recently published by our laboratory [60].

### Analysis of sRNA sequencing data

The adapter sequence was removed using cutadapt (v1.16) [61]. Reads that were 18 nt–30 nt and contain no ambiguous nucleotide (‘N’) were retained as clean reads, and were clustered into unique reads with an in-house python script. For rice sRNA datasets, clean reads were mapped to the ncRNA reference (ftp://ftp.ensemblgenomes.org/pub/plants/release-31/fasta/oryza_sativa/ncrna/Oryza_sativa.IRGSP-1.0.31.ncrna.fa.gz) downloaded from ensemble database to remove reads mapped to known rice rRNAs, tRNAs, snRNAs using bowtie software [62] with perfect matching. For planthopper sRNA datasets, clean reads mapped to the rRNAs, tRNAs, snoRNAs, snRNAs resolved by our lab [60] with perfect matching were discarded. The remaining sRNA reads were then mapped to the host genome (rice or planthopper genome) with perfect matching or to RSV genome, allowing up to one mismatch. Only sRNAs with at least one mapping location were regarded as genuine sRNAs and retained for down-stream analyses. EdgeR [63, 64] was used to identify differentially expressed sRNAs between RSV-infected rice or planthopper samples and the corresponding mock-inoculated samples using 2-fold change in expression and FDR < 0.05 as significance cutoffs. sRNAs with a match to the RSV genome (and without a match to the host genome) were regarded as vsiRNAs. We used psRobot [65] to identify potential target genes of vsiRNAs in rice, and used Miranda [66] to identify potential target genes of vsiRNAs in the planthopper.

### Analysis of mRNA sequencing data

The quality of raw RNA sequencing reads was evaluated with FastQC (http://www.bioinformatics.babraham.ac.uk/projects/fastqc). To restrict to clean reads, low quality regions and adapter sequences were removed using SolexaQA (v2.2) [67] and cutadapt (v1.16), respectively. Reads shorter than 25 bases were discarded. Clean reads were mapped to the host (rice or planthopper, depending on the sample source) genome sequence using HISAT2 [68]. Read counts of the annotated genes were summarized by HTSeq-count [69]. EdgeR [63] was used to identify differentially expressed genes. The genes with at least a 2-fold change in expression level and an FDR < 0.05 were considered to be differentially expressed. GO term enrichment analysis was performed using the R package TopGO [70]. Adrian Alexi’s improved weighted scoring algorithm and Fisher’s test were used to determine the significance of GO term enrichment. The significantly enriched GO terms were inferred with a cutoff *p*-value less than 0.05.

### Reverse transcription PCR for vsiRNAs

Total RNAs was isolated from the viruliferous and nonviruliferous rice/planthoppers using TRIzol Reagent (Invitrogen, Carlsbad, CA, USA). The poly (A) was added to the 3′ end of sRNAs by *E. coli* Poly(A) Polymerase (NEB, Ipswich, MA, USA). The first-strand cDNAs of sRNAs were synthesized using Moloney murine leukemia virus (M-MLV) reverse transcriptase (NEB). The vsiRNAs were subjected to reverse transcription PCR (RT-PCR) using the SYBR Green sRNA expression assays. RT-PCR was amplified for 25–28 cycles. U6 snRNA was amplified as endogenous control. The RT-PCR primers are listed in Additional file 18.

## Conclusions

In summary, we report here on virus/host interactions through vsiRNA-mRNA potential regulation in RSV-infected rice and planthoppers. We have demonstrated the possibility of vsiRNA-mediated downregulation of host genes using qPCR. Thus, it may be possible to use vsiRNA as effector molecules of silencing in artificial miRNA-mediated antiviral resistance. Further studies are needed to verify this interpretation.

## Additional files

Additional file 1: **Table S1.** Summary of sRNA sequencing data in mock-inoculated and RSV-infected rice samples. (XLSX 10 kb)

Additional file 2: **Table S2.** Summary of sRNA sequencing data in mock-inoculated and RSV-infected planthopper samples. (XLSX 13 kb)

Additional file 3: **Figure S1.** Size distribution of sRNAs in the mock-inoculated rice samples (A) and the second replicate of RSV-infected rice sample (B). Numbers of sRNAs with the same size but different 5′ terminal nucleotides were drawn in different colors. (XLS 28 kb)

Additional file 4: **Table S3.** GC content of sRNAs in planthopper and rice samples. (PDF 220 kb)

Additional file 5: **Figure S2.** Size distribution of sRNAs in the mock-inoculated planthopper samples (A) and other two replicates of RSV-infected planthopper samples (B). Numbers of sRNAs with the same size but different 5′ terminal nucleotides were drawn in different colors. (PDF 298 kb)

Additional file 6: **Figure S3.** 5′ terminal nucleotide frequency of host-derived sRNAs in the mock-inoculated rice samples (A), the RSV-infected rice samples (B), the mock-inoculated planthopper samples (C), and the RSV-infected planthopper samples (D). (PDF 790 kb)

Additional file 7: **Figure S4.** Size distribution of vsiRNAs in the second replicate of RSV-infected rice sample (A) and other two replicates of RSV-infected planthopper samples (B). Numbers of vsiRNAs with the same size but different 5′ terminal nucleotides were drawn in different colors. (PDF 306 kb)

Additional file 8: **Figure S5.** Comparison of size distributions of vsiRNAs in RSV-infected rice and planthopper samples. (PDF 107 kb)

Additional file 9: **Figure S6.** 5′ terminal nucleotide frequency of vsiRNAs in two RSV-infected rice samples (A) and three RSV-infected planthopper samples (B). (PDF 378 kb)

Additional file 10: **Figure S7.** Percentage of vsiRNAs that were derived from the RSV genomic (+) and antigenomic (−) sequences in RSV-infected rice and small brown planthopper samples. (PDF 78 kb)

Additional file 11: **Figure S8.** Number of up- and down-regulated genes in RSV-infected rice and planthopper samples compared with the corresponding mock-inoculated samples. (PDF 102 kb)

Additional file 12: **Figure S9.** Volcano plot to show the fold change and error rates of differentially expressed genes (DEGs) in RSV-infected rice. Green dots represent DEGs. (PDF 118 kb)

Additional file 13: **Figure S10.** Gene ontology (GO) enrichment analysis of the up-regulated genes in RSV-infected rice. (PDF 207 kb)

Additional file 14: **Figure S11.** Volcano plot to show the fold change and error rates of differentially expressed genes (DEGs) in small brown planthopper after infected with RSV for 5 days. Green dots represent DEGs. (PDF 111 kb)

Additional file 15: **Figure S12.** Gene ontology (GO) enrichment analysis for the up-regulated genes in small brown planthopper after infected with RSV for 5 days. (PDF 306 kb)

Additional file 16: **Table S4.** List of 17 down-regulated rice genes that are potentially targeted by rice-specific vsiRNAs. (XLS 34 kb)

Additional file 17: **Table S5.** List of 6 down-regulated planthopper genes that are potentially targeted by planthopper-specific vsiRNAs. (XLSX 54 kb)

Additional file 18: **Table S6.** Primers used for RT-PCR analysis of vsiRNAs in RSV-infected rice and RSV-infected planthopper. (XLS 34 kb)

## Abbreviations

AGO: Argonaute
CMV: *Cucumber mosaic virus*
DEGs: differentially expressed genes
GO: Gene Ontology
M-MLV: Moloney murine leukemia virus
NCBI: National Center for Biotechnology Information
*rdr*s: RNA-dependent RNA polymerases
RSV: *Rice stripe virus*
RT-PCR: reverse transcription PCR
sRNA: small RNA
vsiRNAs: virus-derived small interfering RNAs

## References

[1] Soosaar JL, Burch-Smith TM, Dinesh-Kumar SP (2005). Mechanisms of plant resistance to viruses. Nat Rev Microbiol.

[2] Ng JC, Perry KL (2004). Transmission of plant viruses by aphid vectors. Mol Plant Pathol.

[3] Power AG (2000). Insect transmission of plant viruses: a constraint on virus variability. Curr Opin Plant Biol.

[4] Hohn T (2007). Plant virus transmission from the insect point of view. Proc Natl Acad Sci U S A.

[5] Ding SW (2010). RNA-based antiviral immunity. Nat Rev Immunol.

[6] Gilbertson Robert L., Batuman Ozgur, Webster Craig G., Adkins Scott (2015). Role of the Insect SupervectorsBemisia tabaciandFrankliniella occidentalisin the Emergence and Global Spread of Plant Viruses. Annual Review of Virology.

[7] Ramirez BC, Haenni AL (1994). Molecular biology of tenuiviruses, a remarkable group of plant viruses. J Gen Virol.

[8] de Haro LA, Dumon AD, Mattio MF, Arguello Caro EB, Llauger G, Zavallo D, Blanc H, Mongelli VC, Truol G, Saleh MC (2017). Mal de Rio Cuarto virus infection triggers the production of distinctive viral-derived siRNA profiles in wheat and its Planthopper vector. Front Plant Sci.

[9] Ghoshal B, Sanfacon H (2015). Symptom recovery in virus-infected plants: revisiting the role of RNA silencing mechanisms. Virology.

[10] Ramesh SV, Williams S, Kappagantu M, Mitter N, Pappu HR (2017). Transcriptome-wide identification of host genes targeted by tomato spotted wilt virus-derived small interfering RNAs. Virus Res..

[11] Ratcliff F, Harrison BD, Baulcombe DC (1997). A similarity between viral defense and gene silencing in plants. Science.

[12] Huang J, Yang ML, Zhang XM (2016). The function of small RNAs in plant biotic stress response. J Integr Plant Biol.

[13] Donaire L, Wang Y, Gonzalez-Ibeas D, Mayer KF, Aranda MA, Llave C (2009). Deep-sequencing of plant viral small RNAs reveals effective and widespread targeting of viral genomes. Virology.

[14] Dunoyer P, Voinnet O (2005). The complex interplay between plant viruses and host RNA-silencing pathways. Curr Opin Plant Biol.

[15] Xia Z, Zhao Z, Chen L, Li M, Zhou T, Deng C, Zhou Q, Fan Z (2016). Synergistic infection of two viruses MCMV and SCMV increases the accumulations of both MCMV and MCMV-derived siRNAs in maize. Sci Rep.

[16] Li L, Guo C, Wang B, Zhou T, Lei Y, Dai YH, He W, Liang C, Wang XF (2016). RNAi-mediated transgenic rice resistance to Rice stripe virus. J Integr Agr.

[17] Huang J, Yang ML, Lu L, Zhang XM. Diverse functions of small RNAs in different plant-pathogen communications. Front Microbiol. 2016;7:1552. 10.3389/fmicb.2016.01552 PMC5048074 27757103

[18] Ding SW, Voinnet O (2007). Antiviral immunity directed by small RNAs. Cell.

[19] Mlotshwa S, Pruss GJ, Vance V (2008). Small RNAs in viral infection and host defense. Trends Plant Sci.

[20] Du Peng, Wu Jianguo, Zhang Jiayao, Zhao Shuqi, Zheng Hong, Gao Ge, Wei Liping, Li Yi (2011). Viral Infection Induces Expression of Novel Phased MicroRNAs from Conserved Cellular MicroRNA Precursors. PLoS Pathogens.

[21] Xu Y, Huang L, Fu S, Wu J, Zhou X (2012). Population diversity of rice stripe virus-derived siRNAs in three different hosts and RNAi-based antiviral immunity in Laodelphgax striatellus. PLoS One.

[22] Bouche N, Lauressergues D, Gasciolli V, Vaucheret H (2006). An antagonistic function for Arabidopsis DCL2 in development and a new function for DCL4 in generating viral siRNAs. EMBO J.

[23] Deleris A, Gallego-Bartolome J, Bao J, Kasschau KD, Carrington JC, Voinnet O (2006). Hierarchical action and inhibition of plant dicer-like proteins in antiviral defense. Science.

[24] Fusaro AF, Matthew L, Smith NA, Curtin SJ, Dedic-Hagan J, Ellacott GA, Watson JM, Wang MB, Brosnan C, Carroll BJ (2006). RNA interference-inducing hairpin RNAs in plants act through the viral defence pathway. EMBO Rep.

[25] Galiana-Arnoux D, Dostert C, Schneemann A, Hoffmann JA, Imler JL (2006). Essential function in vivo for Dicer-2 in host defense against RNA viruses in drosophila. Nat Immunol.

[26] van Rij RP, Saleh MC, Berry B, Foo C, Houk A, Antoniewski C, Andino R (2006). The RNA silencing endonuclease Argonaute 2 mediates specific antiviral immunity in Drosophila melanogaster. Genes Dev.

[27] Wang XH, Aliyari R, Li WX, Li HW, Kim K, Carthew R, Atkinson P, Ding SW (2006). RNA interference directs innate immunity against viruses in adult Drosophila. Science.

[28] Falk BW, Tsai JH (1998). Biology and molecular biology of viruses in the genus Tenuivirus. Annu Rev Phytopathol.

[29] Xiong RY, Wu JX, Zhou YJ, Zhou XP (2008). Identification of a movement protein of the Tenuivirus Rice stripe virus. J Virol.

[30] Li J, Xiang CY, Yang J, Chen JP, Zhang HM (2015). Interaction of HSP20 with a viral RdRp changes its sub-cellular localization and distribution pattern in plants. Sci Rep.

[31] Wei TY, Yang JG, Liao FR, Gao FL, Lu LM, Zhang XT, Li F, Wu ZJ, Lin QY, Xie LH (2009). Genetic diversity and population structure of rice stripe virus in China. J Gen Virol.

[32] Shi BB, Lin L, Wang SH, Guo Q, Zhou H, Rong LL, Li JM, Peng JJ, Lu YW, Zheng HY (2016). Identification and regulation of host genes related to Rice stripe virus symptom production. New Phytol.

[33] Wang W, Zhao W, Li J, Luo L, Kang L, Cui F. The c-Jun N-terminal kinase pathway of a vector insect is activated by virus capsid protein and promotes viral replication. Elife. 2017;6:e26591. 10.7554/eLife.26591 PMC5515582 28716183

[34] Yan F, Zhang H, Adams MJ, Yang J, Peng J, Antoniw JF, Zhou Y, Chen J (2010). Characterization of siRNAs derived from rice stripe virus in infected rice plants by deep sequencing. Arch Virol.

[35] Angell SM, Baulcombe DC (1997). Consistent gene silencing in transgenic plants expressing a replicating potato virus X RNA. EMBO J.

[36] Miozzi L, Gambino G, Burgyan J, Pantaleo V (2013). Genome-wide identification of viral and host transcripts targeted by viral siRNAs in Vitis vinifera. Mol Plant Pathol.

[37] Qi X, Bao FS, Xie Z (2009). Small RNA deep sequencing reveals role for Arabidopsis thaliana RNA-dependent RNA polymerases in viral siRNA biogenesis. PLoS One.

[38] Shimura Hanako, Pantaleo Vitantonio, Ishihara Takeaki, Myojo Nobutoshi, Inaba Jun-ichi, Sueda Kae, Burgyán József, Masuta Chikara (2011). A Viral Satellite RNA Induces Yellow Symptoms on Tobacco by Targeting a Gene Involved in Chlorophyll Biosynthesis using the RNA Silencing Machinery. PLoS Pathogens.

[39] Smith NA, Eamens AL, Wang MB (2011). Viral small interfering RNAs target host genes to mediate disease symptoms in plants. PLoS Pathog.

[40] Wang J, Tang Y, Yang Y, Ma N, Ling X, Kan J, He Z, Zhang B (2016). Cotton leaf curl Multan virus-derived viral small RNAs can target cotton genes to promote viral infection. Front Plant Sci.

[41] Diaz-Pendon JA, Li F, Li WX, Ding SW (2007). Suppression of antiviral silencing by cucumber mosaic virus 2b protein in Arabidopsis is associated with drastically reduced accumulation of three classes of viral small interfering RNAs. Plant Cell.

[42] Mi SJ, Cai T, Hu YG, Chen Y, Hodges E, Ni FR, Wu L, Li S, Zhou H, Long CZ (2008). Sorting of small RNAs into Arabidopsis argonaute complexes is directed by the 5 ' terminal nucleotide. Cell.

[43] Montgomery TA, Howell MD, Cuperus JT, Li DW, Hansen JE, Alexander AL, Chapman EJ, Fahlgren N, Allen E, Carrington JC (2008). Specificity of ARGONAUTE7-miR390 interaction and dual functionality in TAS3 trans-acting siRNA formation. Cell.

[44] Khvorova A, Reynolds A, Jayasena SD (2003). Functional siRNAs and miRNAs exhibit strand bias. Cell.

[45] Ghildiyal M, Xu J, Seitz H, Weng ZP, Zamore PD (2010). Sorting of Drosophila small silencing RNAs partitions microRNA* strands into the RNA interference pathway. Rna.

[46] Wang XB, Wu QF, Ito T, Cillo F, Li WX, Chen XM, Yu JL, Ding SW (2010). RNAi-mediated viral immunity requires amplification of virus-derived siRNAs in Arabidopsis thaliana. P Natl Acad Sci USA.

[47] Ho T, Wang H, Pallett D, Dalmay T (2007). Evidence for targeting common siRNA hotspots and GC preference by plant dicer-like proteins. FEBS Lett.

[48] Agrawal S, Kelkenberg M, Begum K, Steinfeld L, Williams CE, Kramer KJ, Beeman RW, Park Y, Muthukrishnan S, Merzendorfer H (2014). Two essential peritrophic matrix proteins mediate matrix barrier functions in the insect midgut. Insect Biochem Mol Biol.

[49] Kato N, Mueller CR, Fuchs JF, Mcelroy K, Wessely V, Higgs S, Christensen BM (2008). Evaluation of the function of a type I Peritrophic matrix as a physical barrier for midgut epithelium invasion by mosquito-borne pathogens in Aedes aegypti. Vector-Borne Zoonot.

[50] Gullner G, Juhasz C, Nemeth A, Barna B (2017). Reactions of tobacco genotypes with different antioxidant capacities to powdery mildew and tobacco mosaic virus infections. Plant Physiol Biochem.

[51] van Loon LC, Rep M, Pieterse CMJ. Significance of inducible defense-related proteins in infected plants. Annu Rev Phytopathol. 2006;44:135–62. 10.1146/annurev.phyto.44.070505.143425 16602946

[52] Huang Y, Li T, Xu ZS, Wang F, Xiong AS (2017). Six NAC transcription factors involved in response to TYLCV infection in resistant and susceptible tomato cultivars. Plant Physiol Biochem.

[53] Huang Y, Li MY, Wu P, Xu ZS, Que F, Wang F, Xiong AS (2016). Members of WRKY group III transcription factors are important in TYLCV defense signaling pathway in tomato (Solanum lycopersicum). BMC Genomics.

[54] Mondal HA, Chakraborti D, Majumder P, Roy P, Roy A, Bhattacharya SG, Das S (2011). Allergenicity assessment of Allium sativum leaf agglutinin, a potential candidate protein for developing sap sucking insect resistant food crops. PLoS One.

[55] Prasad V, Mishra SK, Srivastava S, Srivastava A (2014). A virus inhibitory protein isolated from Cyamopsis tetragonoloba (L.) Taub. Upon induction of systemic antiviral resistance shares partial amino acid sequence homology with a lectin. Plant Cell Rep.

[56] Wang Z, Zhang K, Sun X, Tang K, Zhang J (2005). Enhancement of resistance to aphids by introducing the snowdrop lectin gene gna into maize plants. J Biosci.

[57] Yarasi B, Sadumpati V, Immanni CP, Vudem DR, Khareedu VR (2008). Transgenic rice expressing Allium sativum leaf agglutinin (ASAL) exhibits high-level resistance against major sap-sucking pests. BMC Plant Biol.

[58] Zhao W, Lu LX, Yang PC, Cui N, Kang L, Cui F (2016). Organ-specific transcriptome response of the small brown planthopper toward rice stripe virus. Insect Biochem Molec.

[59] Zhao W, Yang PC, Kang L, Cui F (2016). Different pathogenicities of Rice stripe virus from the insect vector and from viruliferous plants. New Phytol.

[60] Zhu JJ, Jiang F, Wang XH, Yang PC, Bao YY, Zhao W, Wang W, Lu H, Wang QS, Cui N, et al. Genome sequence of the small brown planthopper, Laodelphax striatellus. Gigascience. 2017;6(12):1–12. 10.1093/gigascience/gix109 PMC5740986 29136191

[61] Martin M (2011). Cutadapt removes adapter sequences from high-throughput sequencing reads. EMBnet Journal.

[62] Langmead Ben, Trapnell Cole, Pop Mihai, Salzberg Steven L (2009). Ultrafast and memory-efficient alignment of short DNA sequences to the human genome. Genome Biology.

[63] Robinson MD, McCarthy DJ, Smyth GK (2010). edgeR: a Bioconductor package for differential expression analysis of digital gene expression data. Bioinformatics.

[64] McCarthy DJ, Chen YS, Smyth GK (2012). Differential expression analysis of multifactor RNA-Seq experiments with respect to biological variation. Nucleic Acids Res.

[65] Wu HJ, Ma YK, Chen T, Wang M, Wang XJ (2012). PsRobot: a web-based plant small RNA meta-analysis toolbox. Nucleic Acids Res.

[66] John B, Enright AJ, Aravin A, Tuschl T, Sander C, Marks DS (2004). Human MicroRNA targets. PLoS Biol.

[67] Cox Murray P, Peterson Daniel A, Biggs Patrick J (2010). SolexaQA: At-a-glance quality assessment of Illumina second-generation sequencing data. BMC Bioinformatics.

[68] Kim D, Landmead B, Salzberg SL (2015). HISAT: a fast spliced aligner with low memory requirements. Nat Methods.

[69] Anders S, Pyl PT, Huber W (2015). HTSeq--a Python framework to work with high-throughput sequencing data. Bioinformatics.

[70] Alexa AaR, J. topGO: Enrichment Analysis for Gene Ontology. R package version 2301 2016.

