# Supplementary figures and images for: Rice stripe virus-derived siRNAs play different regulatory roles in rice and in the insect vector Laodelphax striatellus
Source: BMC Plant Biol. 2018 Oct 4;18:219. doi: 10.1186/s12870-018-1438-7 (PMC6172784; doi:10.1186/s12870-018-1438-7)

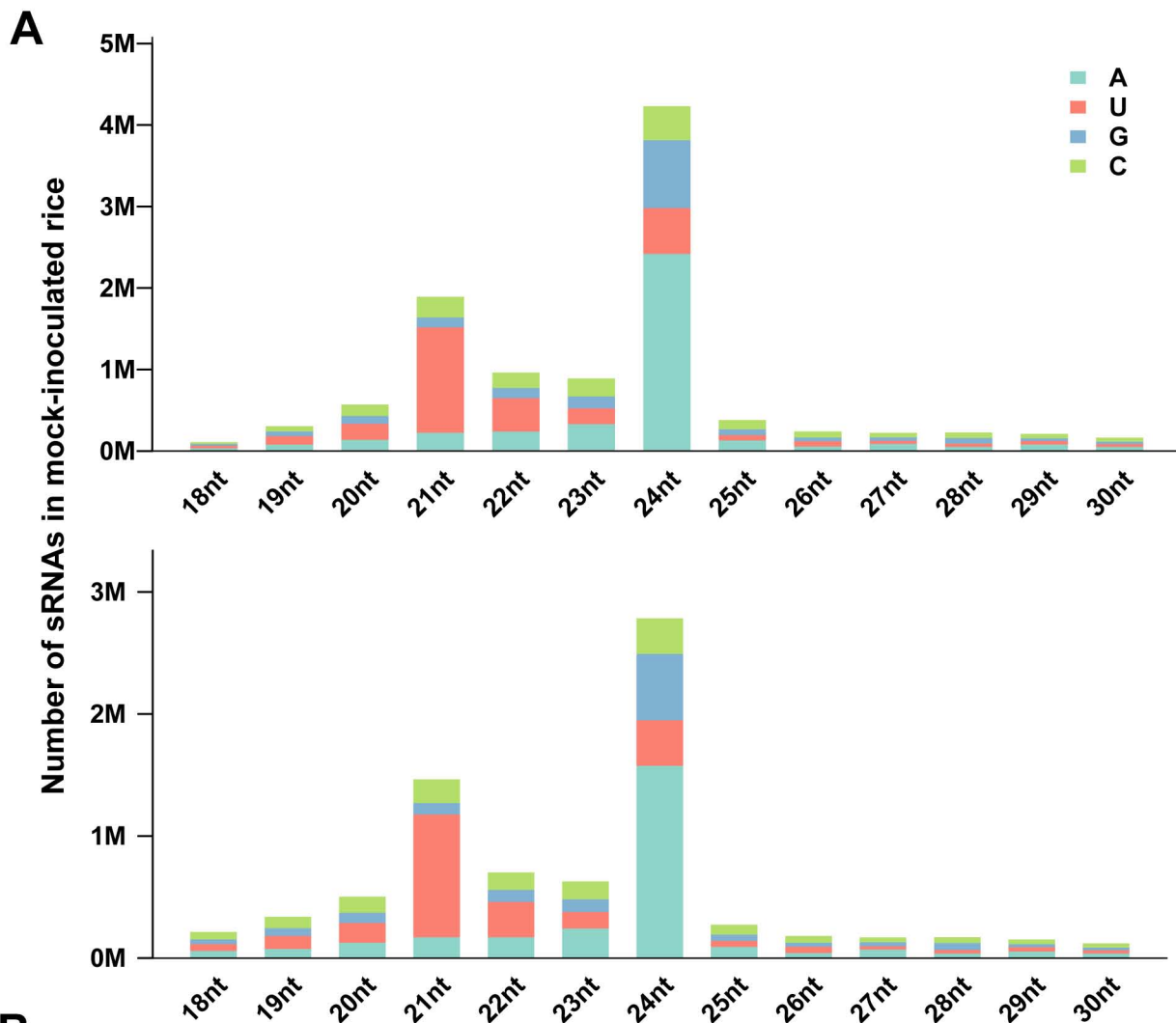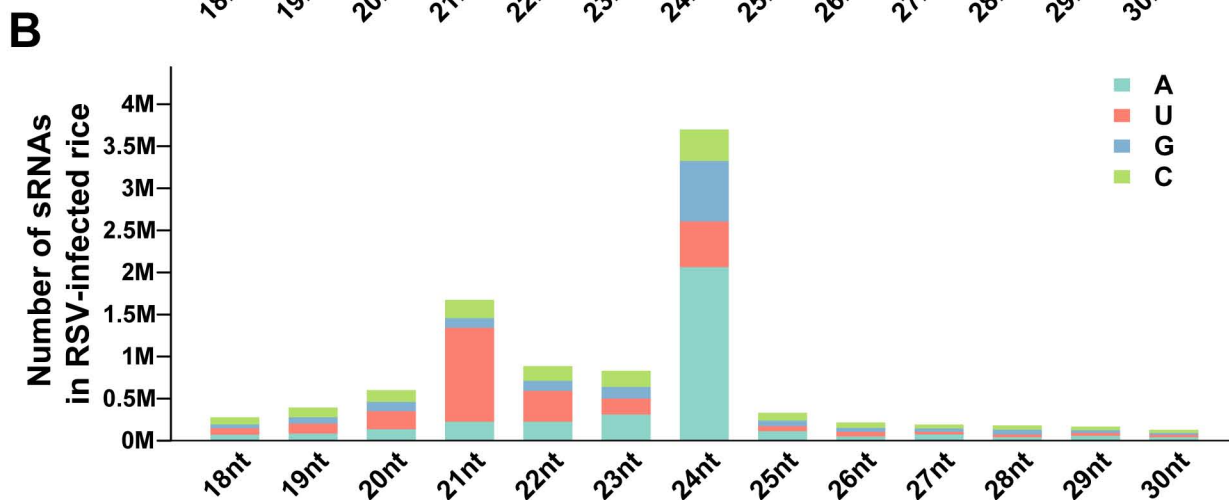

Supplement: Supplementary file 4 — Table S3. GC content of sRNAs in planthopper and rice samples. (PDF 220 kb) [file 12870_2018_1438_MOESM4_ESM.pdf]

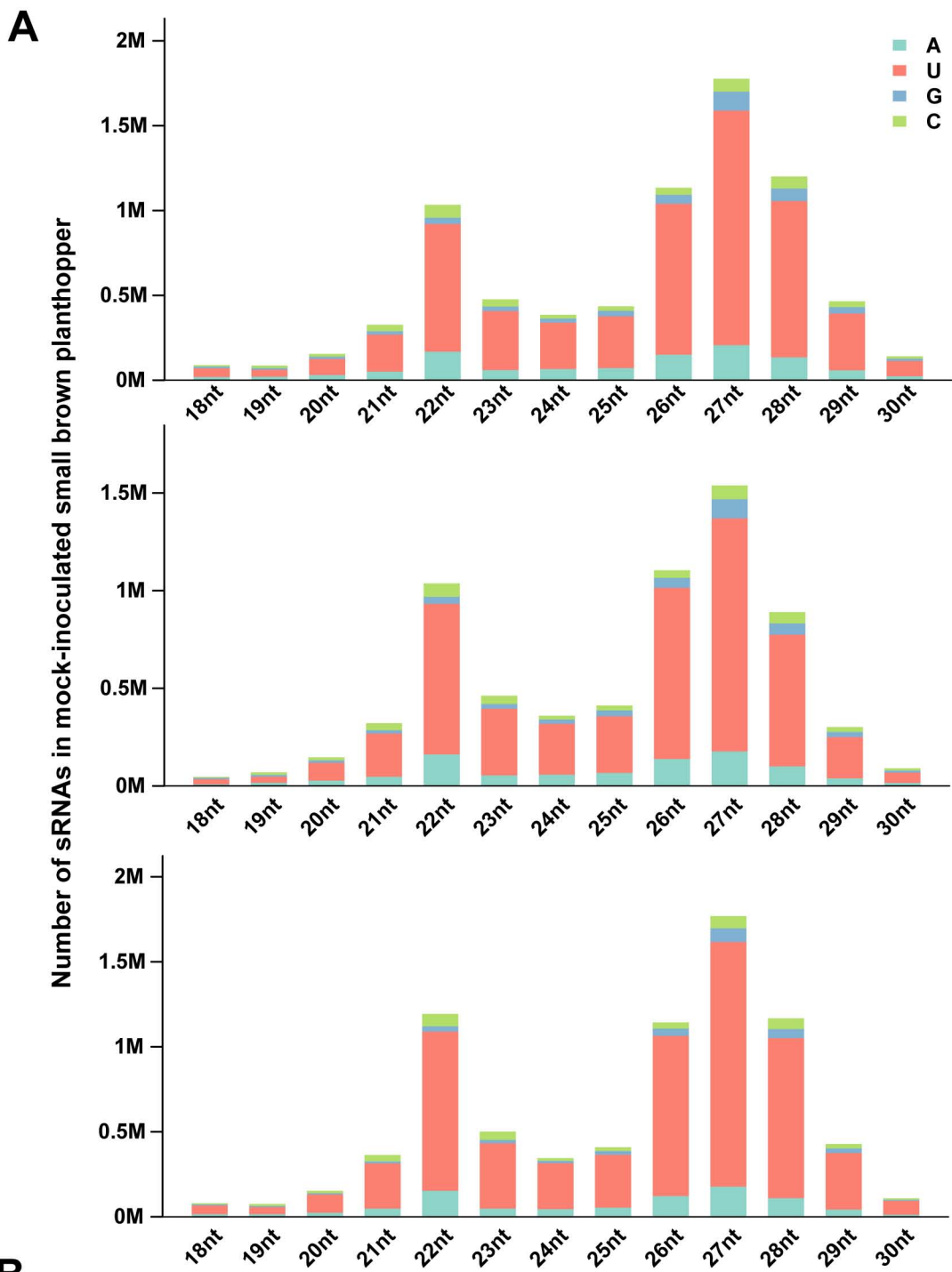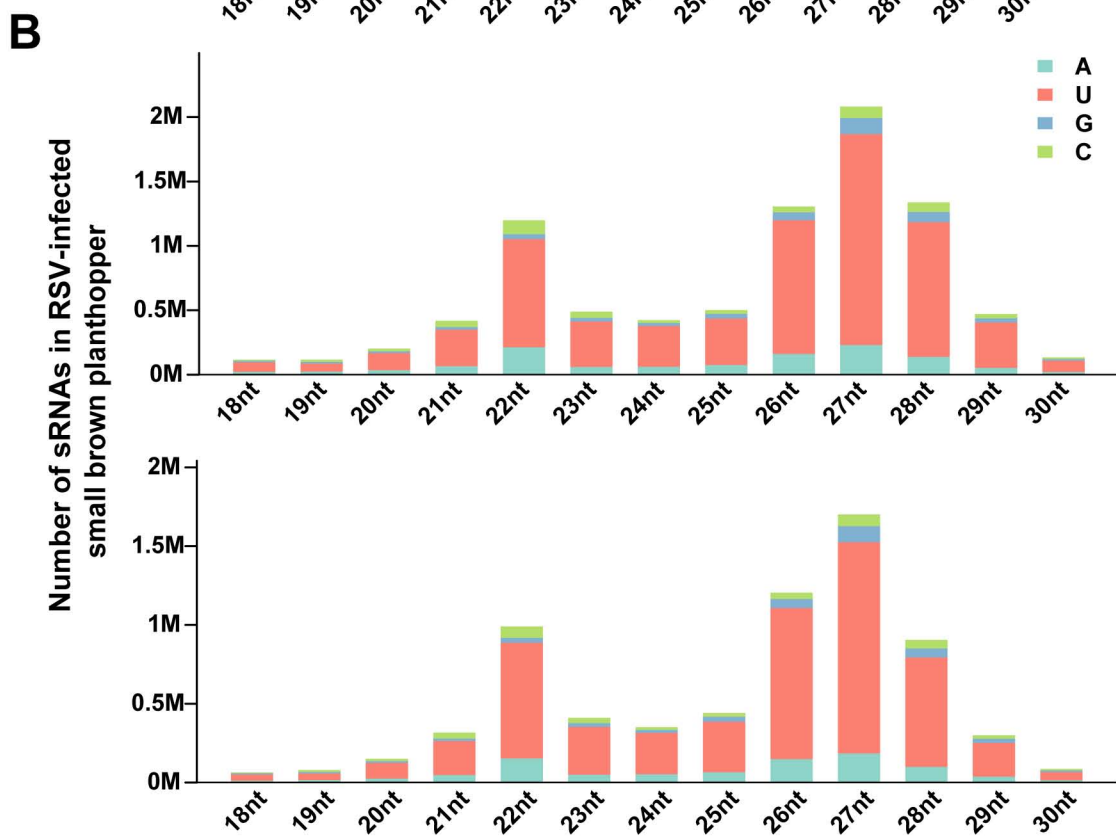

Supplement: Supplementary file 5 — Figure S2. Size distribution of sRNAs in the mock-inoculated planthopper samples (A) and other two replicates of RSV-infected planthopper samples (B). Numbers of sRNAs with the same size but different 5′ terminal nucleotides were drawn in different colors. (PDF 298 kb) [file 12870_2018_1438_MOESM5_ESM.pdf]

**A**

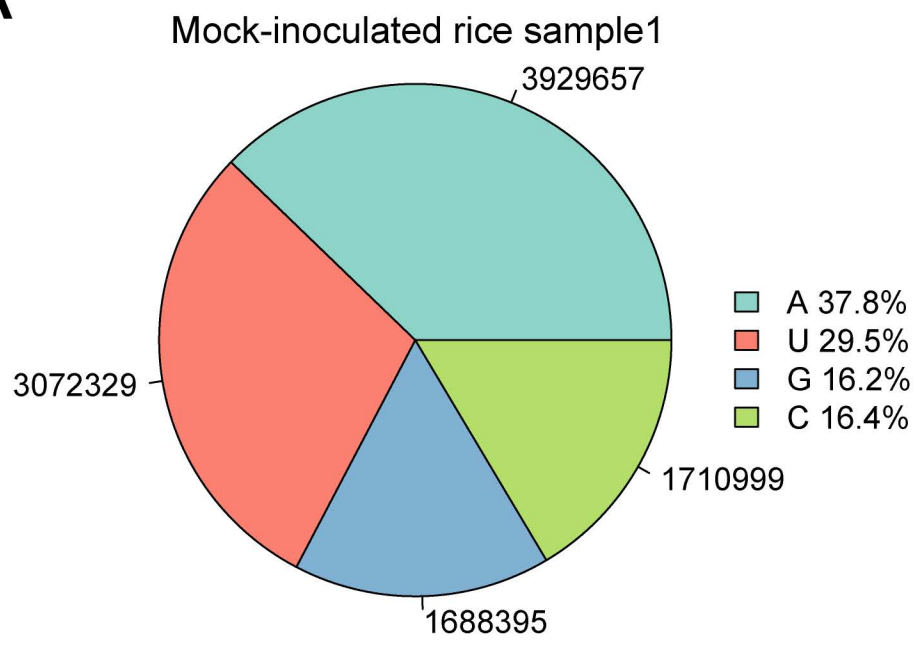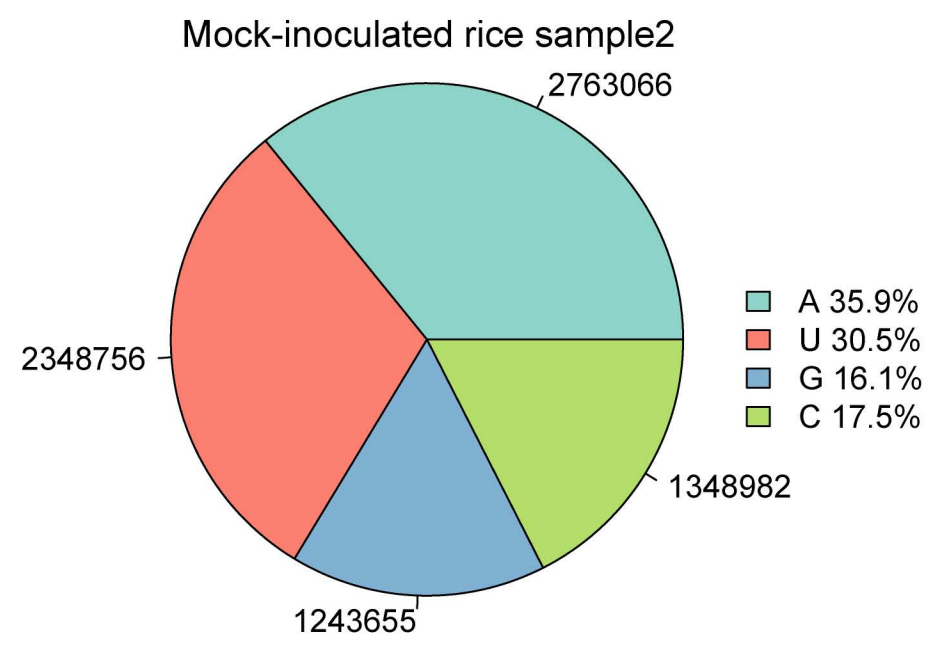

**B**

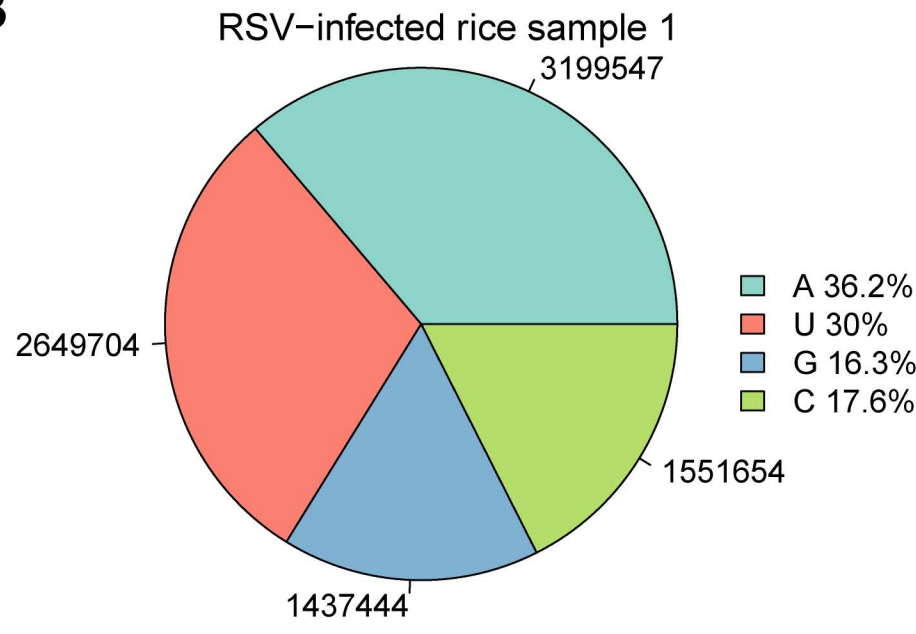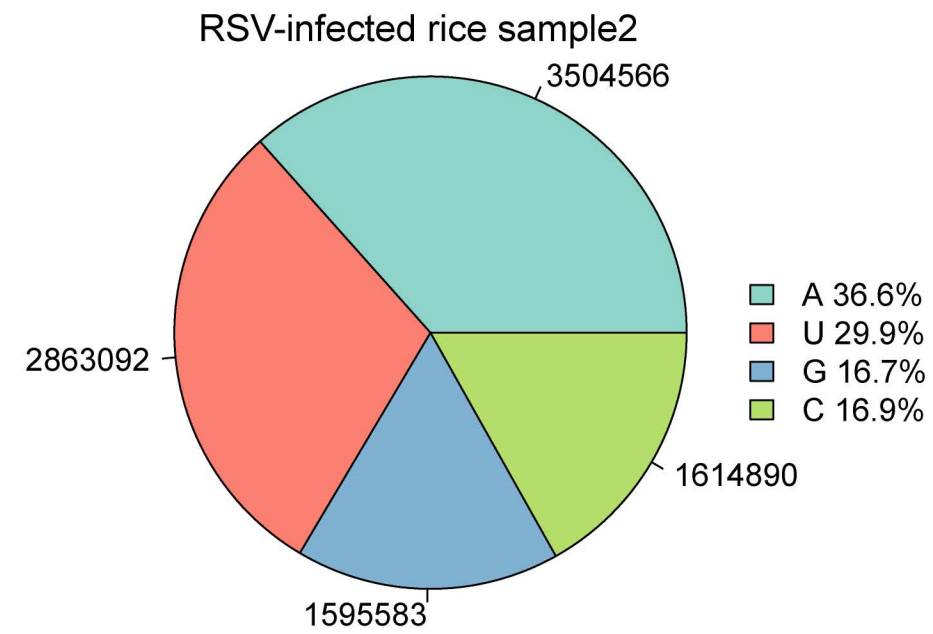

**C**

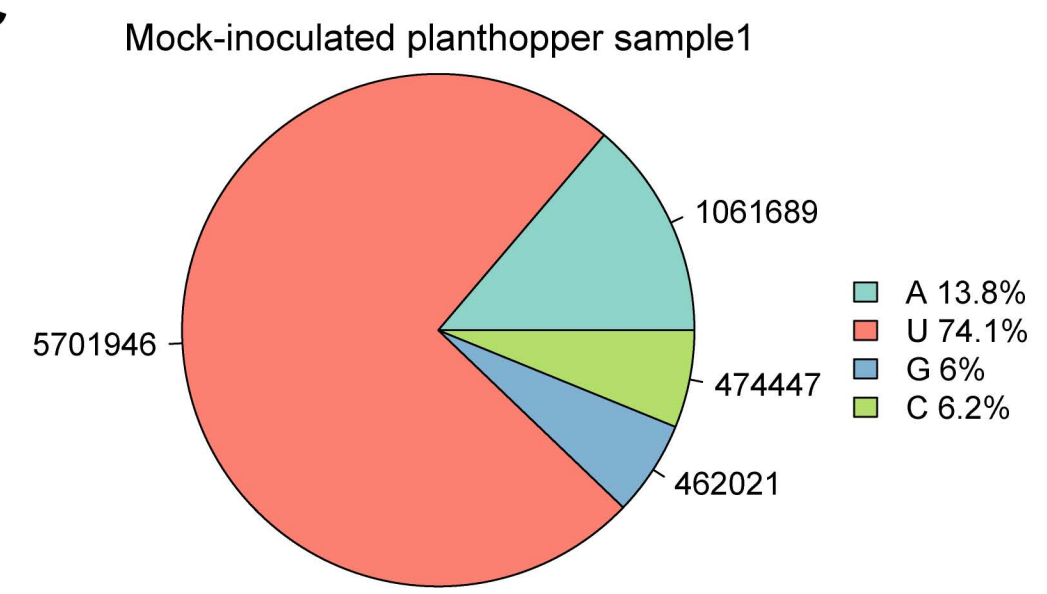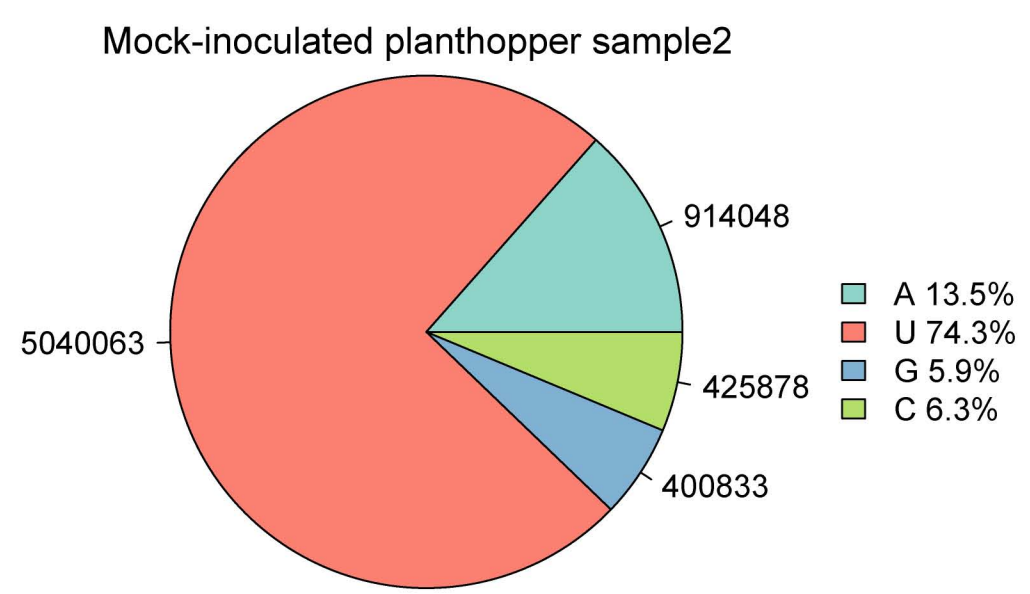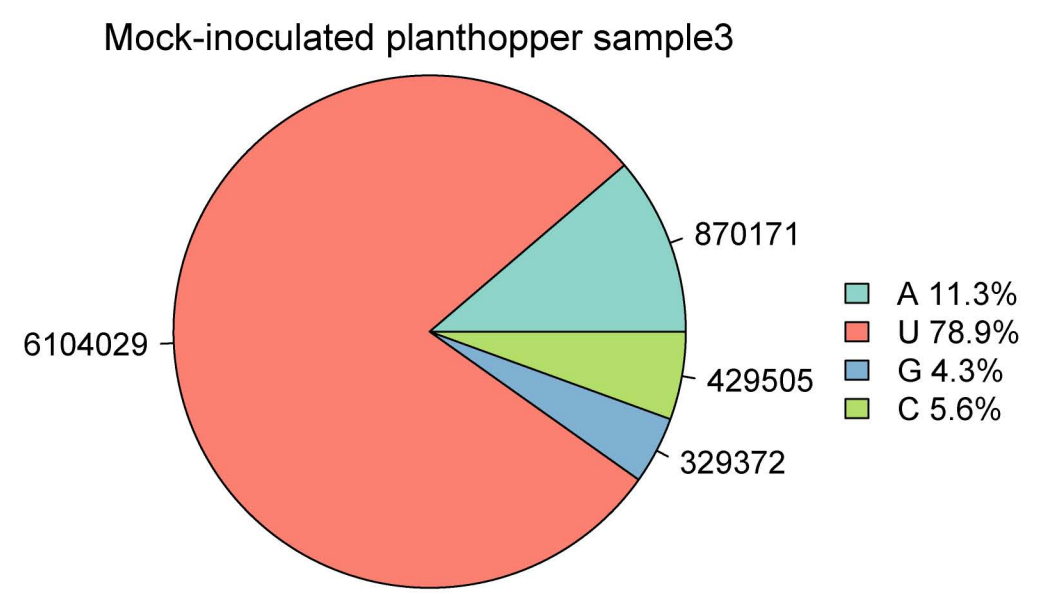

**D**

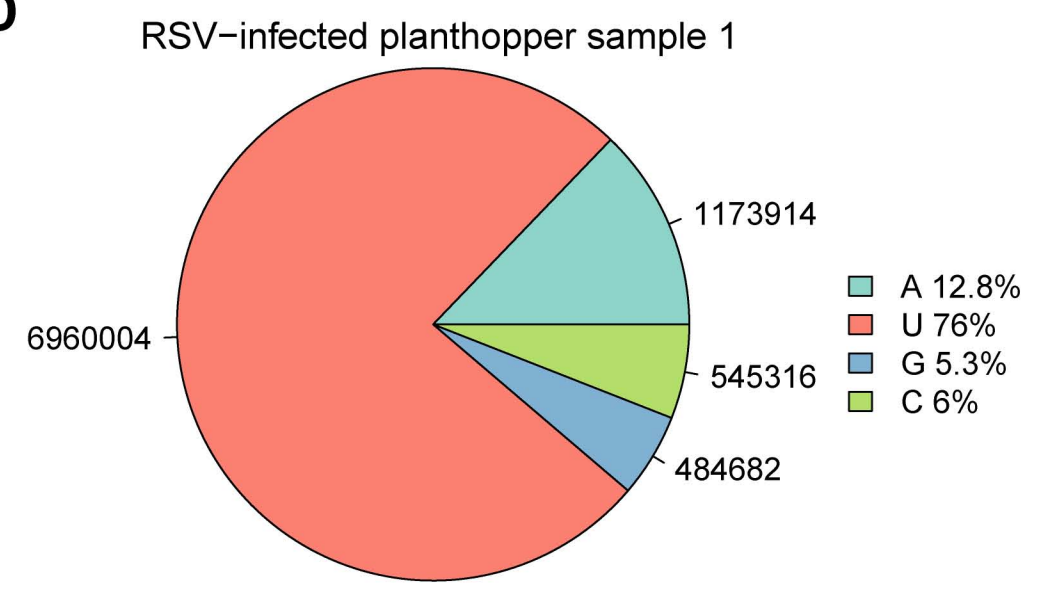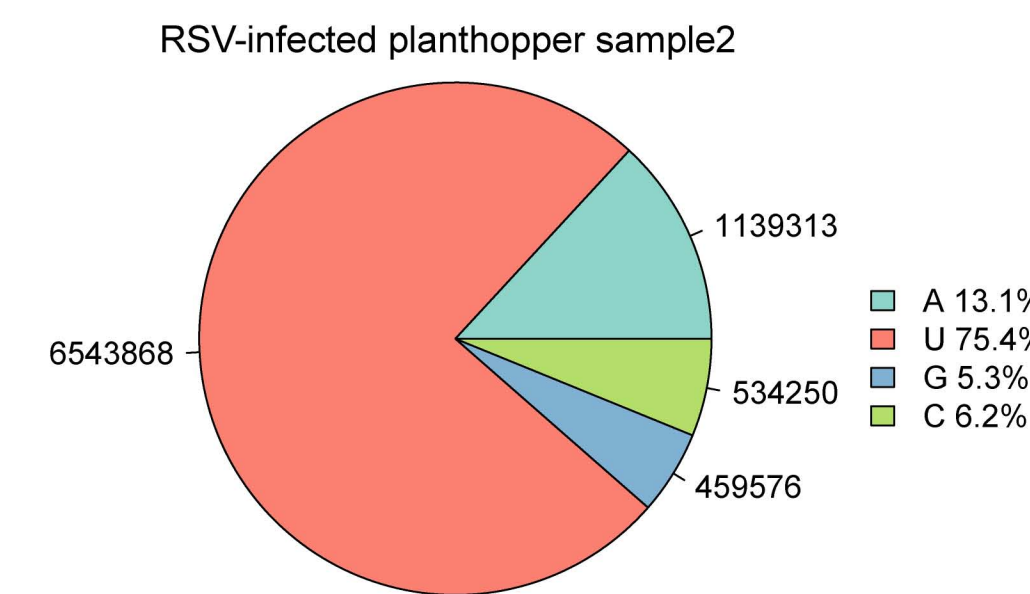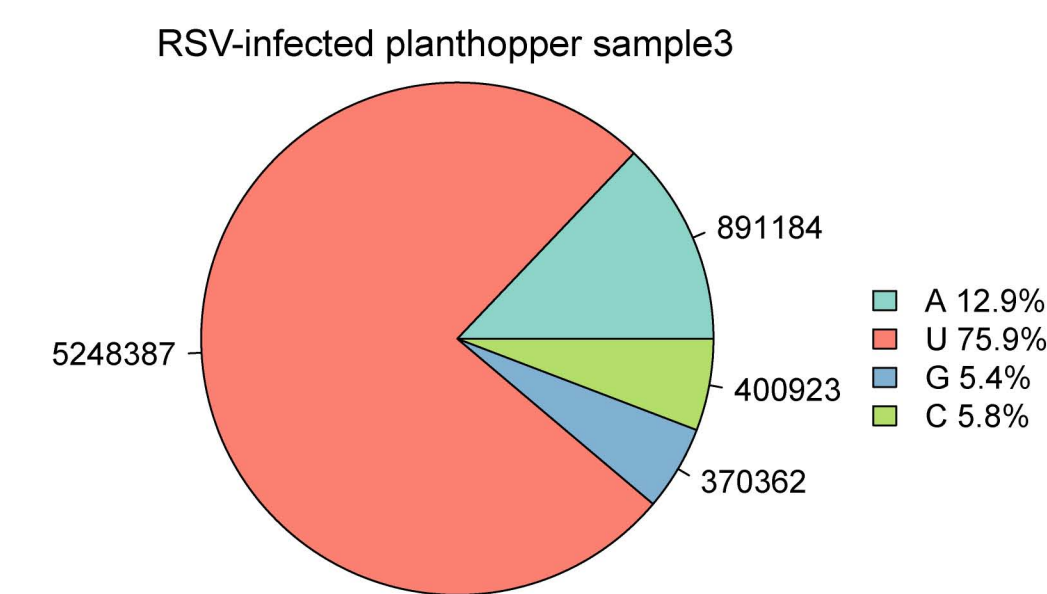

Supplement: Supplementary file 6 — Figure S3. 5′ terminal nucleotide frequency of host-derived sRNAs in the mock-inoculated rice samples (A), the RSV-infected rice samples (B), the mock-inoculated planthopper samples (C), and the RSV-infected planthopper samples (D). (PDF 790 kb) [file 12870_2018_1438_MOESM6_ESM.pdf]

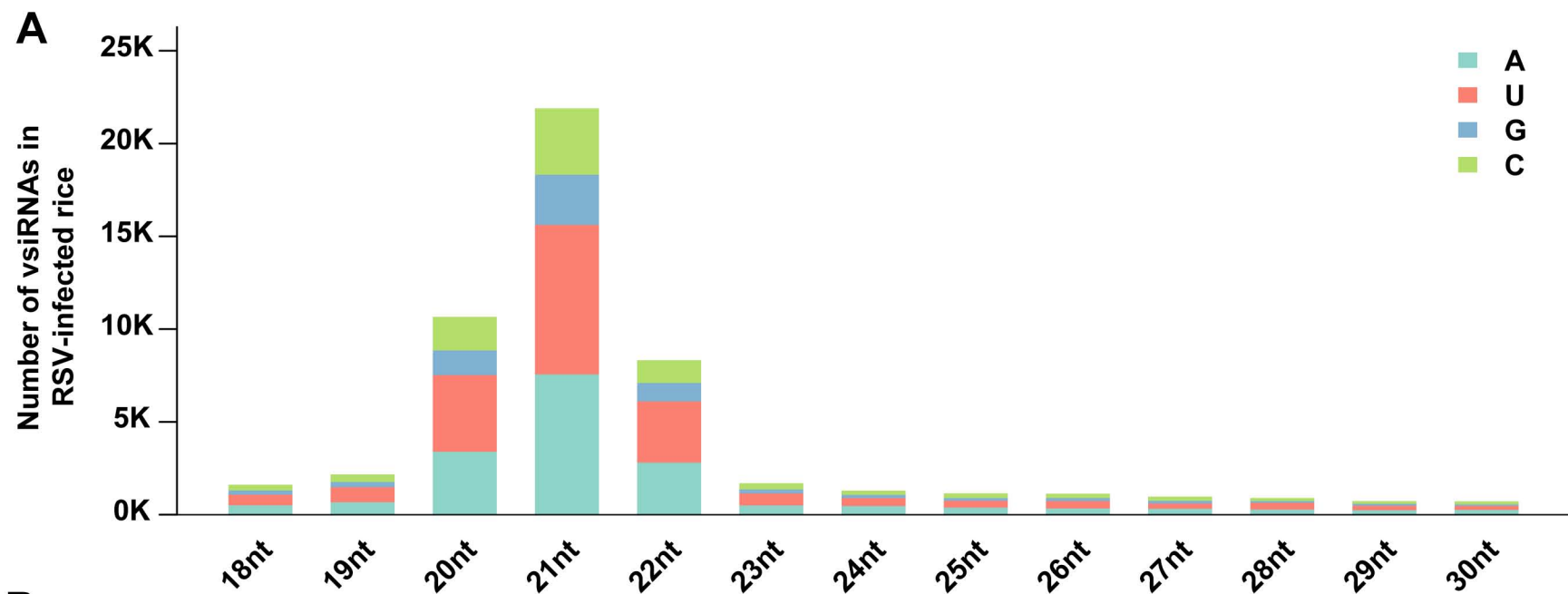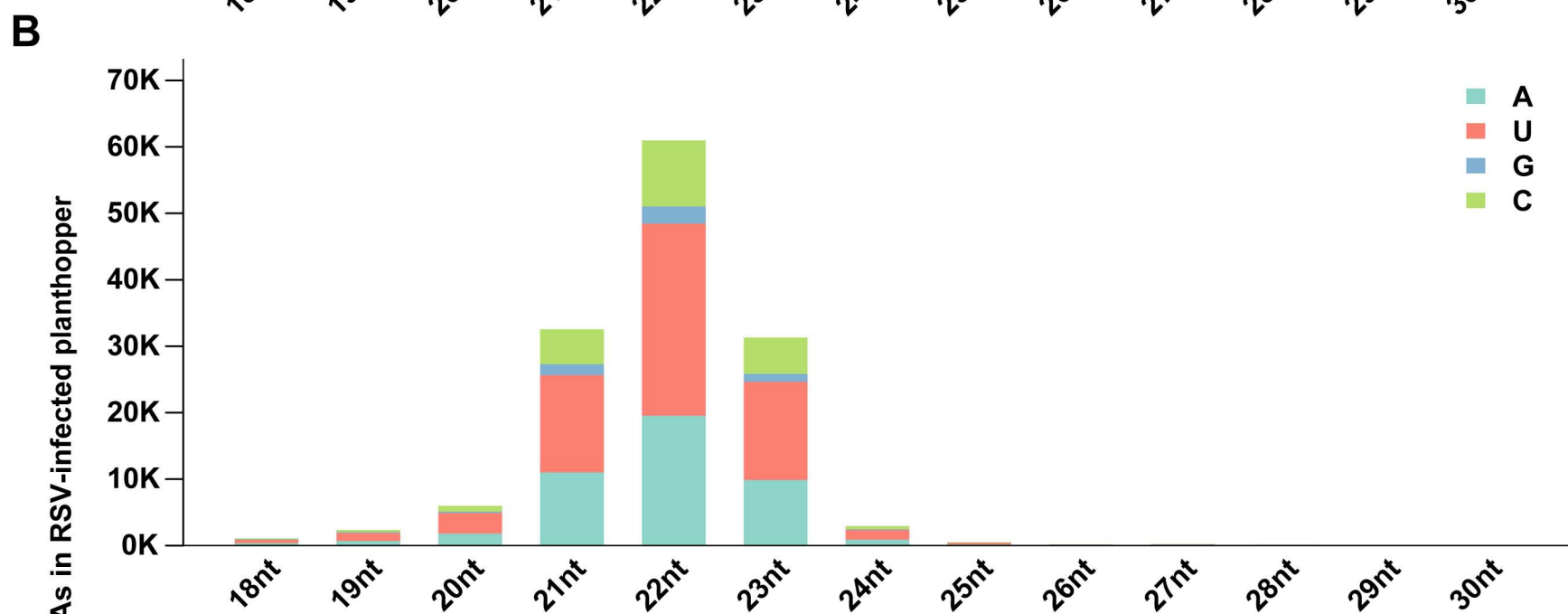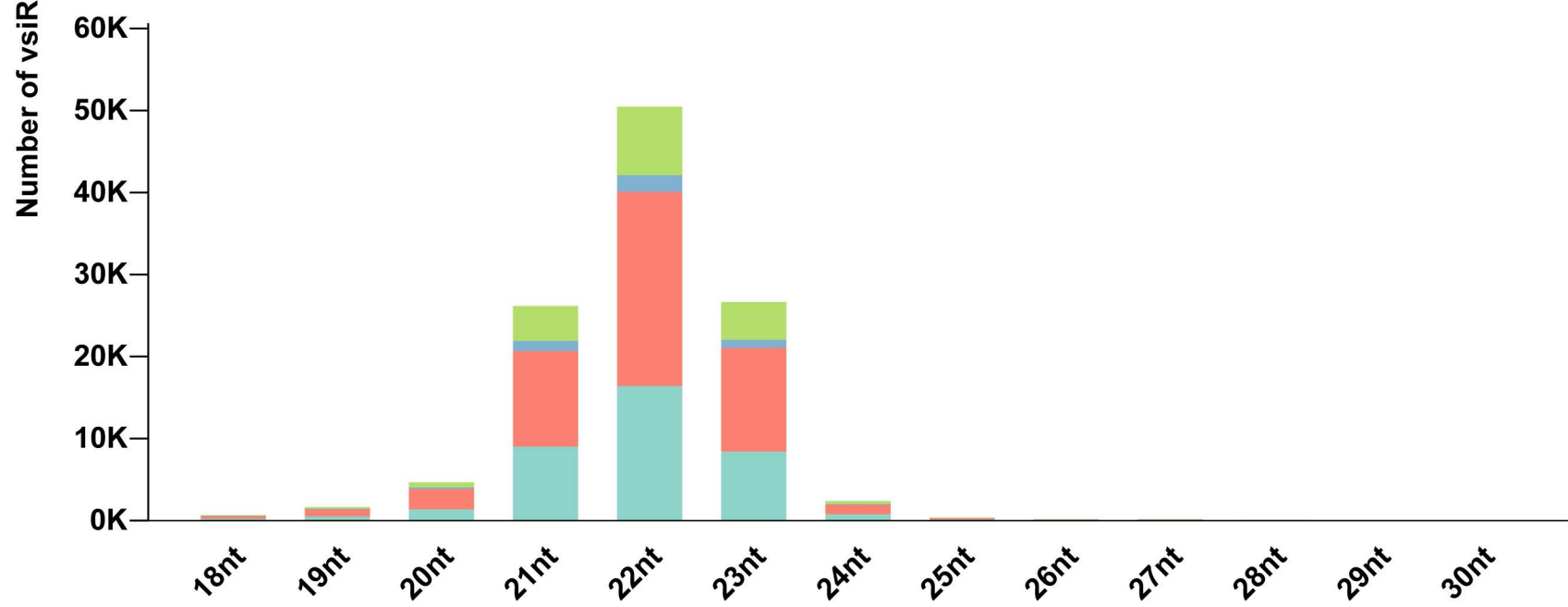

Supplement: Supplementary file 7 — Figure S4. Size distribution of vsiRNAs in the second replicate of RSV-infected rice sample (A) and other two replicates of RSV-infected planthopper samples (B). Numbers of vsiRNAs with the same size but different 5′ terminal nucleotides were drawn in different colors. (PDF 306 kb) [file 12870_2018_1438_MOESM7_ESM.pdf]

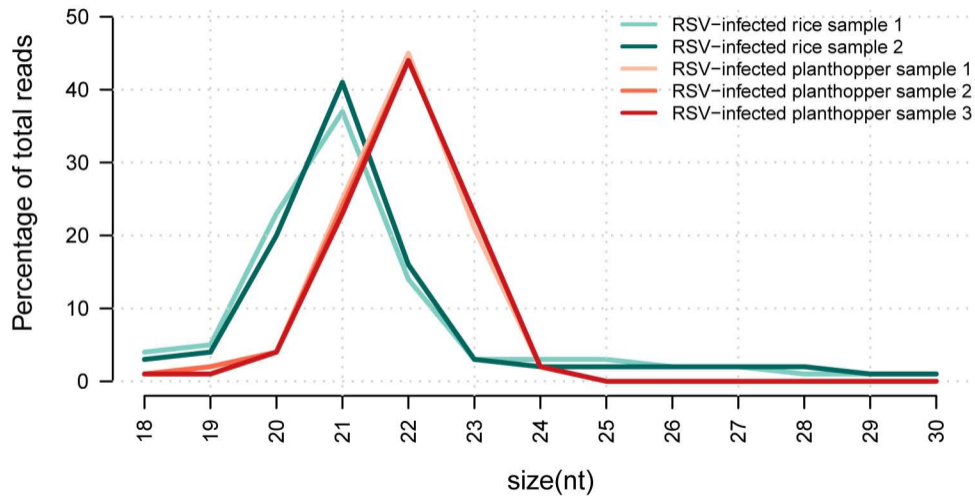

Supplement: Supplementary file 8 — Figure S5. Comparison of size distributions of vsiRNAs in RSV-infected rice and planthopper samples. (PDF 107 kb) [file 12870_2018_1438_MOESM8_ESM.pdf]

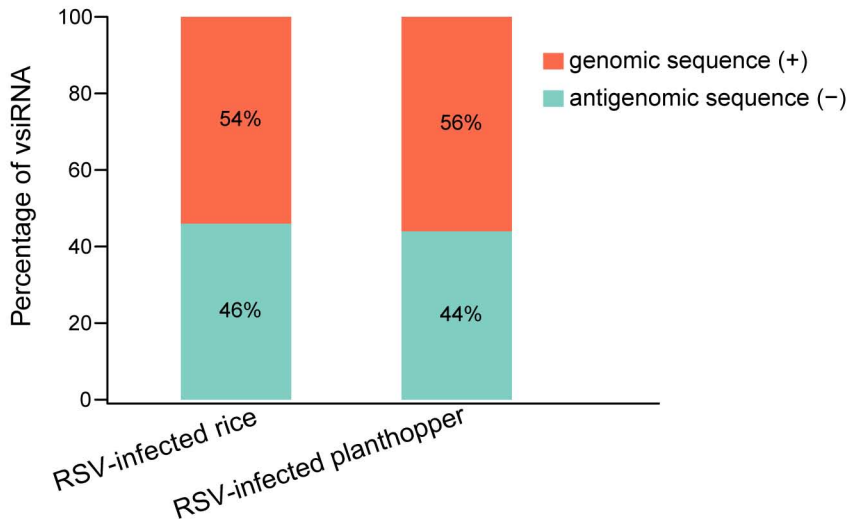

Supplement: Supplementary file 10 — Figure S7. Percentage of vsiRNAs that were derived from the RSV genomic (+) and antigenomic (−) sequences in RSV-infected rice and small brown planthopper samples. (PDF 78 kb) [file 12870_2018_1438_MOESM10_ESM.pdf]

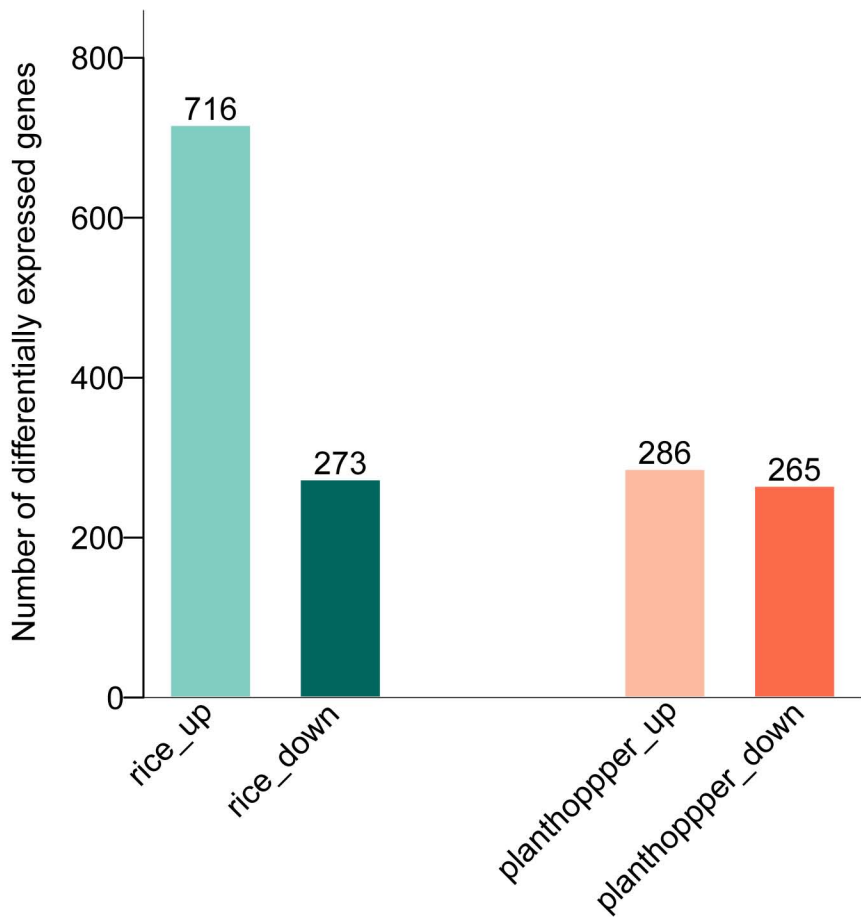

Supplement: Supplementary file 11 — Figure S8. Number of up- and down-regulated genes in RSV-infected rice and planthopper samples compared with the corresponding mock-inoculated samples. (PDF 102 kb) [file 12870_2018_1438_MOESM11_ESM.pdf]

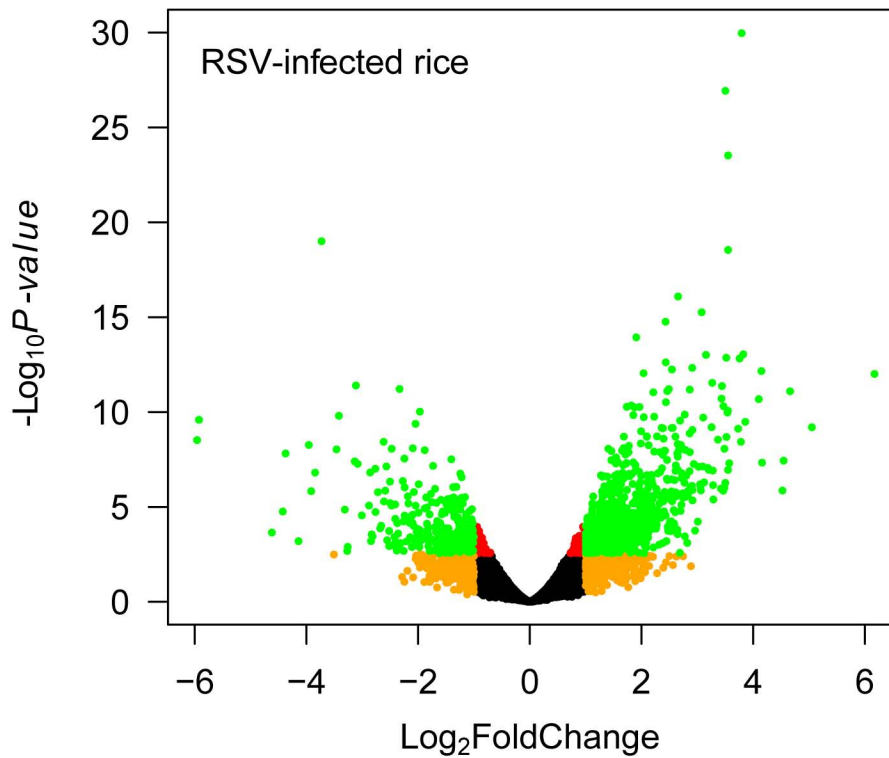

Supplement: Supplementary file 12 — Figure S9. Volcano plot to show the fold change and error rates of differentially expressed genes (DEGs) in RSV-infected rice. Green dots represent DEGs. (PDF 118 kb) [file 12870_2018_1438_MOESM12_ESM.pdf]

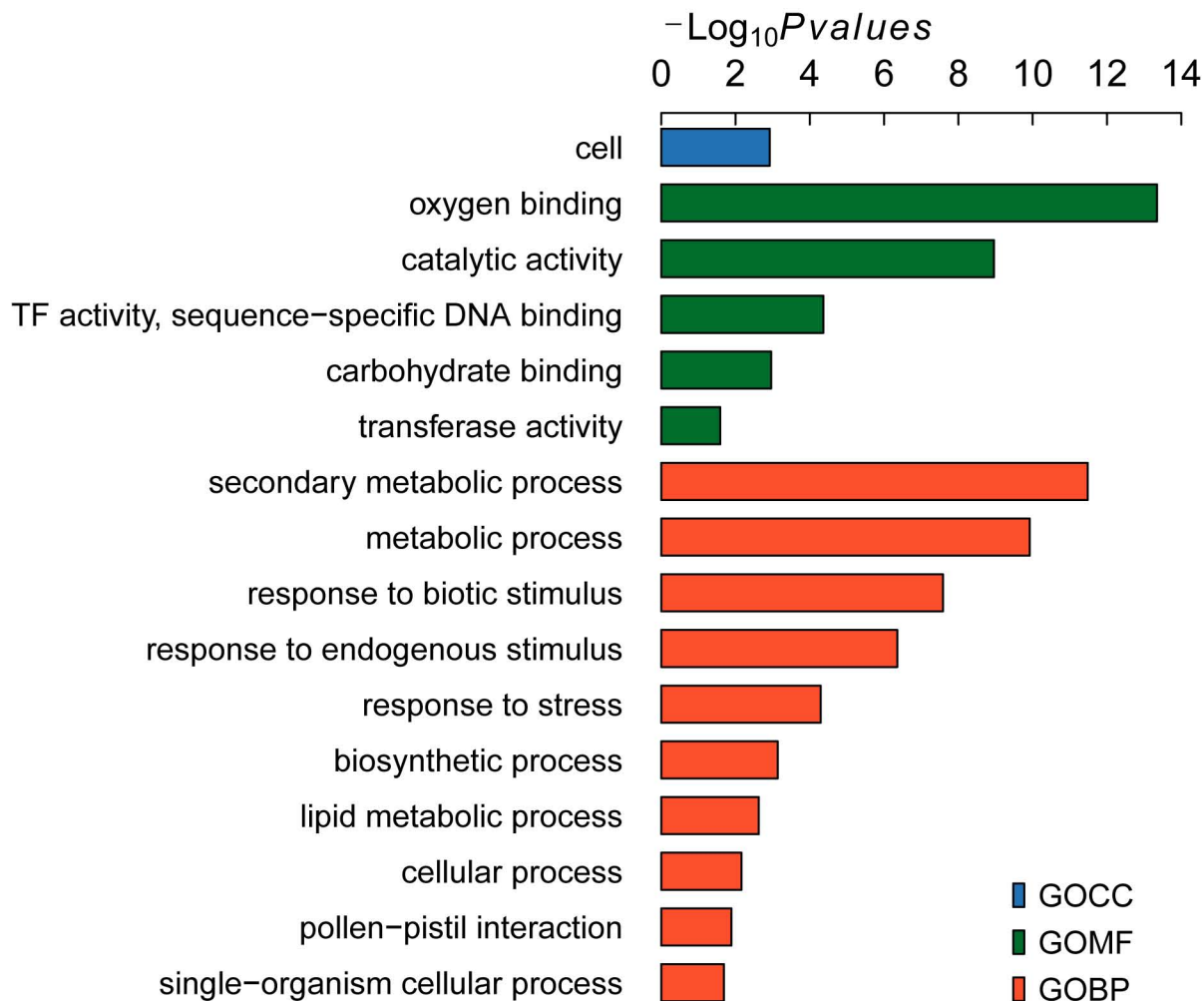

Supplement: Supplementary file 13 — Figure S10. Gene ontology (GO) enrichment analysis of the up-regulated genes in RSV-infected rice. (PDF 207 kb) [file 12870_2018_1438_MOESM13_ESM.pdf]

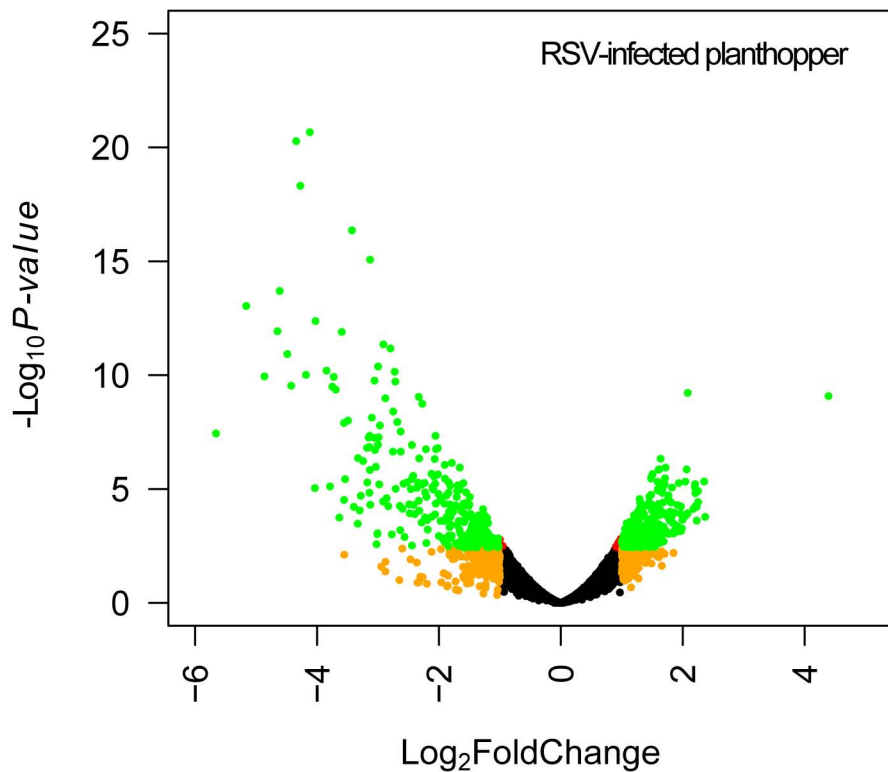

Supplement: Supplementary file 14 — Figure S11. Volcano plot to show the fold change and error rates of differentially expressed genes (DEGs) in small brown planthopper after infected with RSV for 5 days. Green dots represent DEGs. (PDF 111 kb) [file 12870_2018_1438_MOESM14_ESM.pdf]

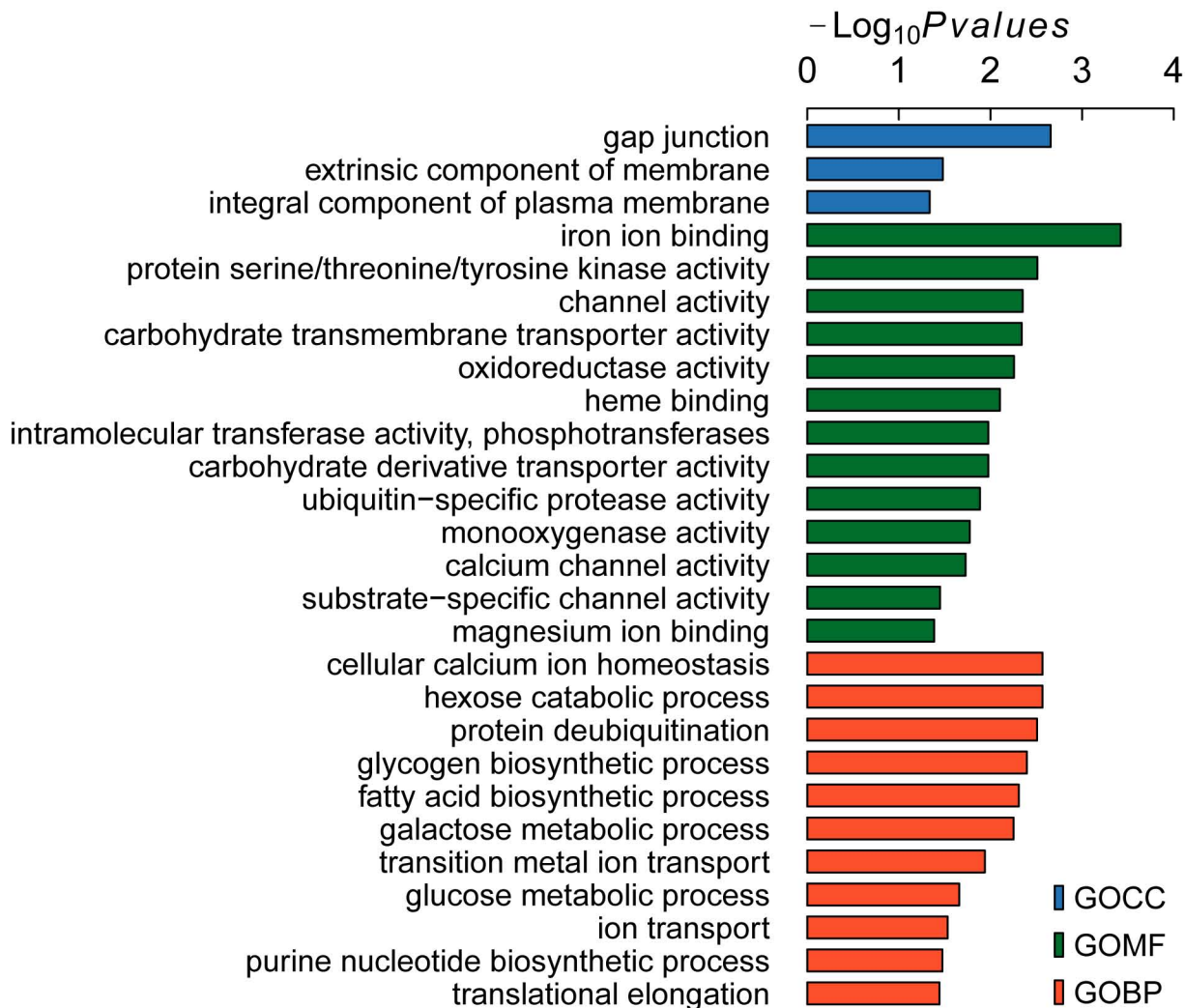

Supplement: Supplementary file 15 — Figure S12. Gene ontology (GO) enrichment analysis for the up-regulated genes in small brown planthopper after infected with RSV for 5 days. (PDF 306 kb) [file 12870_2018_1438_MOESM15_ESM.pdf]
