# Supplementary material for: Rice stripe virus-derived siRNAs play different regulatory roles in rice and in the insect vector Laodelphax striatellus
Source: BMC Plant Biol. 2018 Oct 4;18:219. doi: 10.1186/s12870-018-1438-7 (PMC6172784; doi:10.1186/s12870-018-1438-7)
Supplement: Supplementary file 9 — Figure S6. 5′ terminal nucleotide frequency of vsiRNAs in two RSV-infected rice samples (A) and three RSV-infected planthopper samples (B). (PDF 378 kb) [file 12870_2018_1438_MOESM9_ESM.pdf]

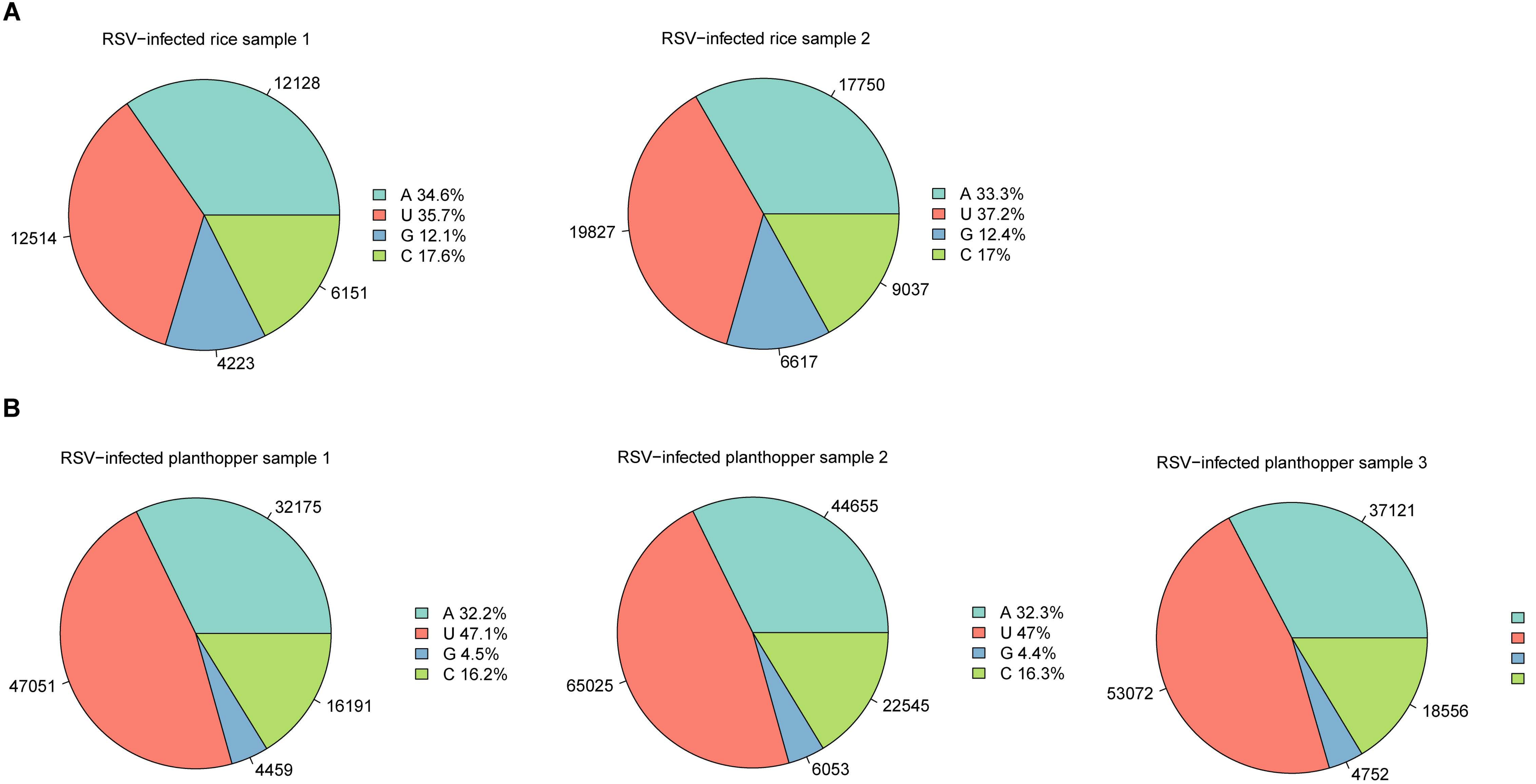
A
RSV−infected rice sample 1
12128
12514
6151
4223
A 34.6%
U 35.7%
G 12.1%
C 17.6%
RSV−infected rice sample 2
17750
19827
9037
6617
A 33.3%
U 37.2%
G 12.4%
C 17%
B
RSV−infected planthopper sample 1
32175
47051
16191
4459
A 32.2%
U 47.1%
G 4.5%
C 16.2%
RSV−infected planthopper sample 2
44655
65025
22545
6053
A 32.3%
U 47%
G 4.4%
C 16.3%
RSV−infected planthopper sample 3
37121
53072
18556
4752
